# Mechanical phenotyping of K562 cells by the Micropipette Aspiration Technique allows identifying mechanical changes induced by drugs

**DOI:** 10.1038/s41598-018-19563-z

**Published:** 2018-01-19

**Authors:** Alessandro Di Cerbo, Valentina Rubino, Francesca Morelli, Giuseppina Ruggiero, Rosaria Landi, Gianandrea Guidetti, Sergio Canello, Giuseppe Terrazzano, Andrea Alessandrini

**Affiliations:** 1 Department of Physics, Informatics and Mathematics, University of Modena and Reggio Emilia, Via G. Campi 213/A, 41125 Modena, Italy; 2 Department of Molecular Medicine and Medical Biotechnology, University of Naples Federico II, Via Pansini 5, 80131 Naples, Italy; 3 Division of Research and Development, SANYpet SpA, Via Austria 3, 35023 Bagnoli di Sopra (PD), Italy; 4 Research and Development Department, Forza10 USA Corp, ORLANDO FL, USA; 5 Department of Science, University of Basilicata, Via Sauro 85, 85100 Potenza, Italy; 6 CNR-Nanoscience Institute- S3, Via Campi 213/A, 41125 Modena, Italy; 7 Department of Translational Medical Sciences, University of Naples Federico II, Via Pansini 16, 80131 Naples, Italy; 8 Department of Molecular Medicine and Medical Biotechnology, University of Naples, Federico II, Via Pansini 5, 80131 Naples, Italy

## Abstract

Mechanical properties of living cells can be used as reliable markers of their state, such as the presence of a pathological state or their differentiation phase. The mechanical behavior of cells depends on the organization of their cytoskeletal network and the main contribution typically comes from the actomyosin contractile system, in both suspended and adherent cells. In the present study, we investigated the effect of a pharmaceutical formulation (OTC – Ossitetraciclina liquida 20%) used as antibiotic, on the mechanical properties of K562 cells by using the Micropipette Aspiration Technique (MAT). This formulation has been shown to increase in a time dependent way the inflammation and toxicity in terms of apoptosis in *in vitro* experiments on K562 and other types of cells. Here we show that by measuring the mechanical properties of cells exposed to OTC for different incubation times, it is possible to infer modifications induced by the formulation to the actomyosin contractile system. We emphasize that this system is involved in the first stages of the apoptotic process where an increase of the cortical tension leads to the formation of blebs. We discuss the possible relation between the observed mechanical behavior of cells aspirated inside a micropipette and apoptosis.

## Introduction

Mechanical properties of living cells are related to their physiological/pathophysiological changes and metabolic states. This relation prompted a plethora of studies devoted to characterizing mechanical properties of single cells and understanding the link between the phenomenological measurement of mechanical properties and the underlying biochemical events. In many cases, altered mechanical properties of cells have been associated with their pathological conditions. Examples are the development of cell metastatic ability, typically associated with a decreased rigidity^1^, malaria disease^2^ and asthma^3^. Different experimental techniques have been exploited to study the mechanical aspects of living cells. Among these techniques there are Atomic Force Microscopy (AFM)^4,5^, Magnetic Twisting Cytometry (MTC)^6^, Micropipette Aspiration Technique (MAT)^7,8^, Particle Tracking Rheology (PTR)^9^ and the Optical Stretching Technique (OST)^10^.

The mechanical properties of living cells are connected to the state and the activity of the cytoskeleton, with dissimilar contributions from different types of cytoskeletal polymer networks and to the viscous properties of the cytoplasm. One of the most important contributions to the mechanical behavior, when techniques like AFM and MAT are used, comes from the actin component together with myosin II. The complex composed by actin and myosin II is indeed responsible for cell contractility. The organization of the actin network is strongly dependent on the state of the cell (such as for the mitotic or apoptotic phase) and its depolymerization in specific conditions could make other cytoskeleton components such as microtubules or intermediate filaments become more relevant in determining the overall mechanical properties^11–13^. When considering the actin/myosin II complex, there is a fundamental difference between adherent and suspended cells. In the former case, the actin/myosin II couple, together with focal adhesion complexes, give rise to stress fibers whose strength is strongly related to the properties of the substrate on which cells are growing and the main contribution to the cell mechanical properties comes from the stress-fibers and the associated pre-stressed state of cells^14,15^. In suspended cells, stress fibers are not present and the acto/myosin II complex is mainly concentrated in the cortical region, just below the membrane, forming many contacts with it. The distinction is also fundamental to selecting the most suitable technique for the experimental cellular analysis. For example, MAT and OST are more suitable for suspended cells whereas AFM is one of the techniques of choice for adherent cells.

Many theoretical models for the mechanics of cells have been introduced in the literature^16–19^. Also in the case of theoretical modeling it is important to distinguish between adherent and suspended cells. In the case of suspended cells, the introduced theoretical models embrace situations in which just viscous contributions are considered with a constant tension coming from the cortical region (liquid drop model) and situations in which elastic contributions together with viscous dissipation are required to reproduce the experimental results^17,20–22^. The model to be adopted strongly depends on the cell type. In the case of hematopoietic cell types, a heterogeneous model including the elastic-viscous region inside the cell and the cortical tension is frequently used, whereas a homogeneous model represented by spring-dashpot elements is usually exploited for non-hematopoietic cells.

In the case of adherent cells a large consensus has been received by the soft-glass rheology model, which manifests itself by a power-law behavior of the cell stiffness as a function of the frequency of the stimulus used to mechanically probe the cell^23,24^. The model establishes the absence of a characteristic relaxation time for cells in favor of a continuous distribution of relaxation times, highlighting the relevance of disorder, rearrangements and metastability conditions for the cytoskeleton. Within the power-law model, cells are characterized by a fluidity parameter, which can vary from 0 (completely elastic behavior) to 1 (completely viscous behavior) and by an elasticity term (the pre-exponential term). The same type of behavior can be equivalently obtained by creep compliance experiments in the time domain instead of the frequency domain (see SI). The power-law model has been found to accurately describe also the behavior of suspended cells^25–27^, which means that the model doesn’t depend on the presence of stress fibers. It has also been found that the effect of drugs on cells could be described by a preserved power-law relation with a variation on the fluidity parameter^27,28^. The possibility of investigating variations of these parameters could offer the opportunity for an early detection of pathological conditions. At the same time it is in principle possible, by exploiting the measurement of the mechanical parameters, to develop drugs aimed at restoring normal mechanical conditions^29^.

Many of the above mentioned experimental techniques lack high-throughput capabilities and, in considering the broad distribution of parameters defining the mechanical properties of even a single cell type, this limitation could represent an obstacle to clearly identify altered mechanical properties. To overcome these limits, techniques based on microfluidic technology have been developed and exploited to obtain a significant statistics for suspended cells^26,30–32^. However, in some cases, specific techniques such as MAT, even if endowed with a lower throughput, could be relevant to provide evidence of particular features in the mechanical behavior of living cells. In fact, one of the main difficulties in modeling cell mechanical properties is due to the active behavior of the cytoskeleton. With long enough time (tens of seconds) the cytoskeleton could actively rearrange to oppose the imposed deformation. In this case biphasic behaviors could be found in the response of a cell to strains and single cell analysis over long time intervals is required^33,34^.

In the present work, we studied by MAT the behavior of the human K562 erythroleukemic cell line, commonly used as *in vitro* tumor model that is able to stimulate the immune-system^35^, affected by the incubation with a pharmaceutical formulation (Ossitetraciclina liquida 20% - in the following we will refer to this formulation as OTC)^36^. The active ingredient of this formulation (oxytetracycline) represents the main employed antibiotic in zootechnical and veterinary practice as feed supplementation to ensure wellness of farmed animals (i.e. poultry, ovine, bovine and swine livestock)^37–40^. Several studies have investigated the potential toxic effects of oxytetracycline and, in particular, the drug showed teratogenic effects^41^ exerting some impacts on immune system^42–45^. In this regard, we recently demonstrated an *in vitro* pro-inflammatory role of OTC and its ability to induce apoptosis in hematopoietic cells in human and dogs^36,46^.

In this context, we raised the hypothesis that, since the actin cytoskeleton and cell mechanics in general are strongly involved in the different phases of the apoptotic process, the Micropipette Aspiration Technique could detect modification in the mechanical behavior, possibly related to apoptosis, of K562 cells exposed to OTC. Considering that the pro-apoptotic effect of OTC is time-dependent, we were also interested in establishing if MAT could be able to detect some changes in the cells for incubation times shorter than the ones needed to detect the effect by the AnnexinV marker. To this aim we characterized by MAT the creep compliance behavior of cells, which had been exposed to OTC for different incubation times. At the same time, cells exposed to OTC for the same incubation times were characterized by using AnnexinV marker to detect the presence of phosphatidylserine in the outer leaflet of the membrane^47^.

## Material And Methods

### Cells and OTC incubation

The cells used were from the human K562 erythroleukemic cell line^35,36^. Cells were cultured in RPMI 1640 culture medium (Gibco BRL, Rockville, MD) at 37 °C in a humidified atmosphere containing 5% CO_2_. OTC (Ossitetraciclina liquida 20%®, TreI, Reggio Emilia, Italy) was used as previously described^36,45^. Briefly, the K562 cells (2.5 × 10^6^/ml) were cultured in RPMI 1640 medium with 10% FCS, with (in the case of treated cells) or without (untreated control cells) 4 μM OTC at 37 °C for different times (6, 9, 12, 15, 16, 18, 24, and 48 h). The untreated K562 cells were used as control of naturally occurring apoptosis (in absence of OTC) in the experiments. 200 μM hydrogen peroxide (H_2_O_2_) was used as a standard positive control of apoptosis in a 5 × 10^5^ K562 cells/mL culture for 1 h at 37 °C, 5% CO_2_.

Apoptosis was assessed by staining the cell membrane-exposed phosphatidylserine with fluorescein isothiocyanate-conjugated (FITC) Annexin V according to the manufacturer’s instructions (Becton Dickinson PharMingen, San Jose, CA) as previously described^48^. Samples were analyzed by flow cytometry by using a two laser-equipped FACS Calibur (Becton Dickinson PharMingen, San Jose, CA) and the CellQuest Analysis Software. Cells undergoing apoptosis were indicated by the percentage of Annexin V-positive cells through FACS analysis.

### Micropipette Aspiration Technique

Capillaries with an external diameter of 1.5 mm were bought from World Precision Instruments (WPI, Sarasota, FL, USA). BSA for glass surface passivation and blebbistatin to block myosin II activity were purchased from Sigma-Aldrich (Sigma-Aldrich, St. Louis, MO, USA). Blebbistatin was diluted in DMSO to have a final concentration of 50 μM, with DMSO concentration less than 0.5% in the chamber where cells are mechanically characterized.

Microaspiration was performed by using pulled borosilicate glass capillaries with a terminal cylindrical shape and an internal diameter in the order of 5–10 μm. It has been shown that results obtained with pipettes with the diameter in this range should be largely independent of the exact pipette radius^25^. Pipettes were fire-polished to ensure good cell-pipette contact and pretreated with BSA (10 mg mL−1) or Surfasil to avoid adhesion between glass and cells. In the case of pretreatment with BSA the pipettes were immersed in the BSA solution for 5 minutes and then they were thoroughly rinsed with distilled water before being filled with the culture medium. In the case of Surfasil pretreatment, the micropipettes were immersed for 5 minutes in a Surfasil solution diluted in toluene; they were subsequently thoroughly washed with toluene and then kept for 10 minutes in the oven at 90 °C. Each pipette was then connected to a pneumatic pressure transducer (Lorenz MPCU-3) to get pressure differences between the internal side of the pipette and the external solution with a sensitivity of 1 mm H_2_O. The pressure difference was applied by controlling the air pressure on top of a cylindrical tube containing the culture medium solution and initially kept at the right height to assure a negligible starting pressure difference (verified by controlling the null aspiration or repulsion of small objects in solution). Cells were kept inside a chamber made by glass-slides separated by a PDMS or Teflon ring allowing the entry of the pipette. We typically changed the chamber containing the cells we were working on every hour taking new cells from the incubator (see Supplementary Material for a discussion on the analysis conditions). The internal sides of the chamber were pretreated with BSA or Surfasil to avoid cell-surface adhesion. We made creep compliance analysis in the time domain (see Supplementary Material). In response to pressure differences (between the internal pipette region and the region just outside the pipette), the cell is aspirated into the pipette and the progressive cell protrusion entry into the pipette can be measured by Optical Microscopy as a function of the applied pressure difference or as a function of time at constant applied pressure. To study the viscoelastic properties of cells, a rapid pressure difference was applied by using a home-developed Lab-view and Arduino-based hardware. After the pressure jump was applied, images of the cell were acquired at a rate of 1 frame per second. Images were acquired by an Olympus IX 70 inverted microscope in Differential Interference Contrast (DIC) mode with a 20x or 40x objective. The images were then analyzed by using the ImageJ software (NIH, Washington, USA) in order to automatically detect the position of the cell protrusion inside the micropipette (a detailed description of the protocol for the automatic detection of the cell protrusion position together with the handling of cell blebs in the analysis is reported in the Supplementary Material). Assuming a linear viscoelastic behavior for the cells, we measured the increase of the projection length starting from the initial position of the projection inside the micropipette. We also verified that the initial holding pressure difference was not able to induce a significant variation of the cell protrusion over times in the order of 3–4 minutes (see Fig. S1).

### Creep compliance analysis

The analysis was based on the power-law model. In this model, the instantaneous creep compliance *J(t)* can be described by:

1 $$J(t)={A}_{J}{(t/{t}_{0})}^{\alpha }$$

where *A*_*J*_ represents the value of *J(t)* for *t* = *t*_0_ = 1 s and α is the power-law exponent. α can vary from 0 (completely elastic behavior) to 1 (completely viscous behavior). The stiffness parameter in Pa can be obtained from the fit of the power-law expression to the relaxation trend^25^:

2 $${A}_{G}=\frac{1}{{A}_{J}{\rm{\Gamma }}(1+\alpha )}$$

where Γ (..) is the gamma function. In some cases we also used the Standard Linear Model or similar models to analyze the cell creep compliance behavior. These models use springs and dashpots to phenomenologically reproduce the experimentally obtained creep behavior of the cells. We found that the power-law model generally fit the behavior in a better way (see Figure S4).

By using the half-space model^49^ it is possible to connect the projection variation inside the pipette to the shear creep compliance to be used for the quantitative fit. According to this model we have:

3 $$J(t)=\frac{2\,\pi }{{{\rm{\Phi }}}_{P}{R}_{P}}\frac{{L}_{p}(t)}{{\rm{\Delta }}P}$$

where *R*_*p*_ is the pipette diameter, Φ_*P*_ is a parameter depending on the ratio between the pipette wall thickness and the pipette radius (typically ≈ 2.1) and Δ*P* is the applied pressure.

If the creep analysis was limited to the first 5 or 6 seconds after the pressure step was applied the behavior of almost all the tested cells could be accounted for by a power-law fit (see below). For longer time-scale several cells deviated significantly from the power-law model. In some cases the cell protrusion inside the micropipette showed a biphasic trend with also oscillatory behavior. In these cases, for different OTC incubation times, we counted the percentages of cells showing the different behaviors. The averaged behaviors for the power-law model at long time scale that we show in this work have been obtained excluding the cells with oscillatory trend. We also considered at short time scale the power-law behavior for cells which undergo an oscillatory behavior at longer time scale.

For each OTC treatment, a set of 20/30 cells was analyzed. Each treatment was also compared to control cells, which were kept for the same time period in normal culture conditions without being exposed to OTC. For the statistical analysis we used the Chi-squared test to establish if the number of cells characterized by each different behavior was different from the situation of the negative control in a statistically significant way. For the statistics of the values of the α-exponent and of the stiffness parameters of the power law behavior we used the Kruskal-Wallis test because in many cases it has been found that the mechanical parameters for a cell population follow a log-normal distribution.

It is important to stress that the diameter of the exploited micropipettes typically exceeds the nucleus size and the measured mechanical properties are not expected to be affected by the mechanics of the nucleus, which is typically more rigid than the cytoplasm and the cortical actin.

## Results

**Figure 1:**
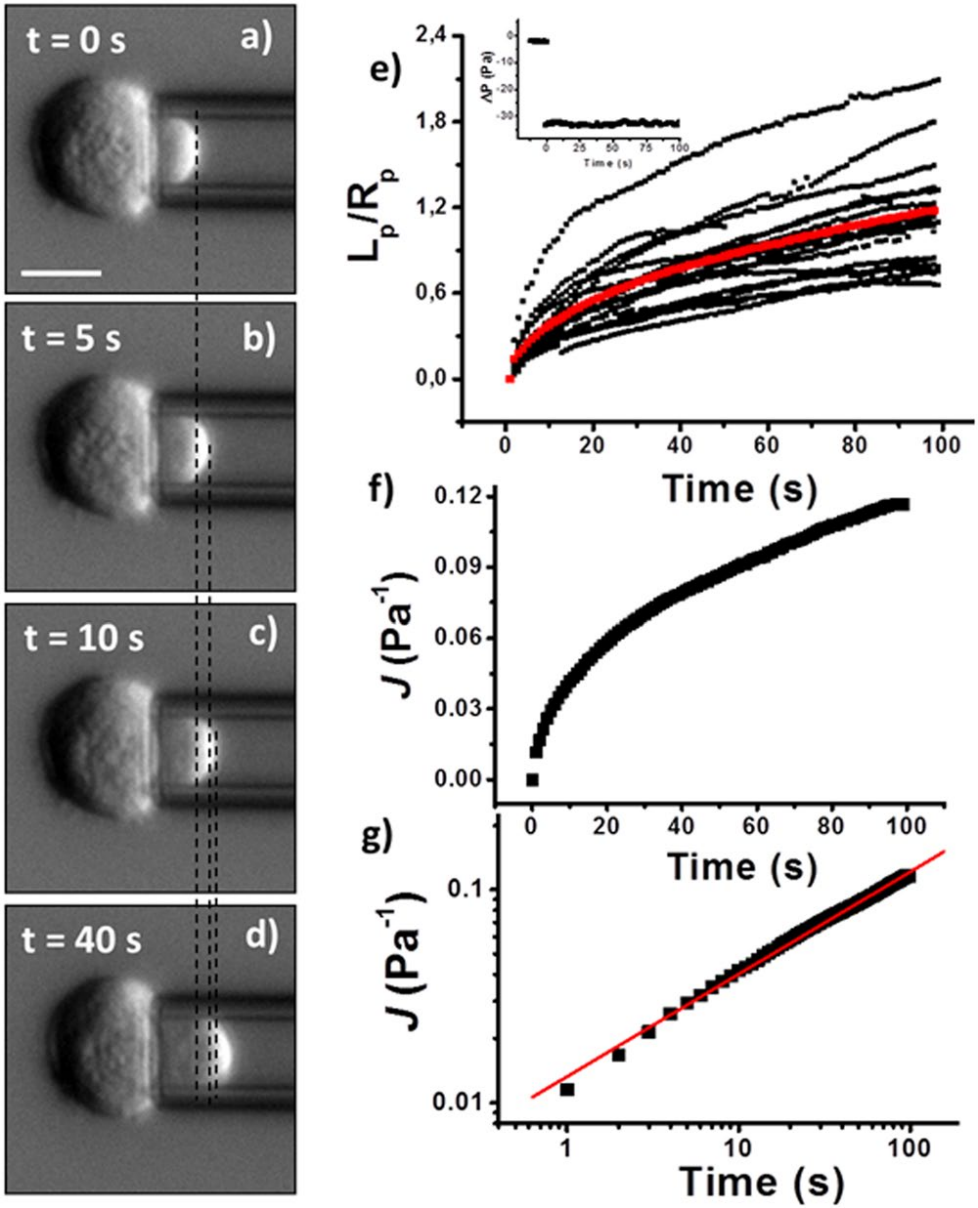
From (**a**) to (**d**): sequence of images (DIC contrast; scale bar =10 μm) of a K562 cell showing the increase of the cell-protrusion length inside the micropipette  after a pressure step of 30 Pa is applied by the micropipette aspiration set-up. The time associated to each image is reported. The dashed black lines have been introduced to mark the position of the cell protrusion in the different snapshots; (**e**) plot showing the cell protrusion variation with time for several cells. The protrusion length variation is normalized to the micropipette radius. The red line represents the averaged behavior. Inset: time variation of the measured pressure difference in the micropipette aspiration set-up. (**f**) plot of the average curve reported in **e**; (**g**) Log-Log representation of the average behavior for the control cells (the same as in **f**) to highlight the power-law fit to the data (continuous red line).

Figure 1 shows the typical behavior of cell protrusion as a function of time for control, not exposed to OTC, K562 cells when a negative pressure jump is applied (see inset to Fig. 1e for the corresponding pressure values). The behavior can be fitted by a power-law relationship with an average feature of the exponent α = 0.44 ± 0.02 (mean ± SEM). In some cases it seems that at short time some experimental points depart from the fit (see Fig. 1g). It is possible that in these cases, at very short time (2–3 s), a power-law regime with a higher fluidity parameter exists, but the fit would be based on a very small number of experimental points. This behavior could be due to the raising ramp of the pressure step or to the cells which are initially not in good contact with the micropipette. In the control sample (considering all the experiments we performed), 32 out of 44 cells showed a creep compliance behavior that could be described by the power-law model. The remaining 12 cells showed a biphasic behavior for the cell projection inside the micropipette. The presence of a retraction movement is related to an active mechanism, which is able to feel the cell deformation and to establish a biochemical mechanism to oppose it, especially at longer time-scales. In some cases there was a fast increase followed by a retraction phase. In these cases the projection comes to an almost equilibrated position or to a minimum followed by a new increase. If cells presenting the latter behavior are kept at the same constant pressure for long periods (about 300 s) an oscillatory movement is observed. In other cases there was a small increase of the projection followed by a continuous decrease. Both types of behavior are represented in Figure S5.

The power-law model has been already applied to describe K562 mechanical properties in the literature^26^. In the case of microfluidic platforms, the entry-time of K562 WT cells in narrow channels has been fitted by a power-law model showing a broad distribution for the exponent value with a peak around a feature of 0.3[26]. The average value we obtained for the exponent is within the broad distribution found from the measurement of the entry–time but it doesn’t correspond to the peak position. The discrepancy could be due to several factors: the different measured phenomenon, the different timescale for the relaxation phenomenon (milliseconds instead of seconds), the different geometry of the channel (square section with respect to cylindrical section of the micropipette), the value of the applied pressure difference (much lower in our case – a few Pa *vs* kPa), and our limited statistical sample. The second parameter we can extract from the fit is the pre-exponential factor. It represents the cell compliance at the reference time t_0_ = 1 s. Its inverse is a measure of the cell stiffness parameter. The average *A*_*J*_ value we obtained for control K562 is (0.0040 ± 0.0015) Pa^−1^ (mean ± SD). Considering the corresponding α value we obtain a feature of about 90 Pa for the stiffness parameter (see Supplementary Material for the details of the used formulas). Similar values have been obtained in other studies using Optical Tweezers^50^ or microfluidic platforms^26^. It is important to stress that we did not perform any type of synchronization on the cell cycle. As a consequence, in the same sample we could have cells in different phases of their life cycle, each one characterized by its mechanical properties resulting in a large standard deviation for the fluidity parameter.

**Figure 2:**
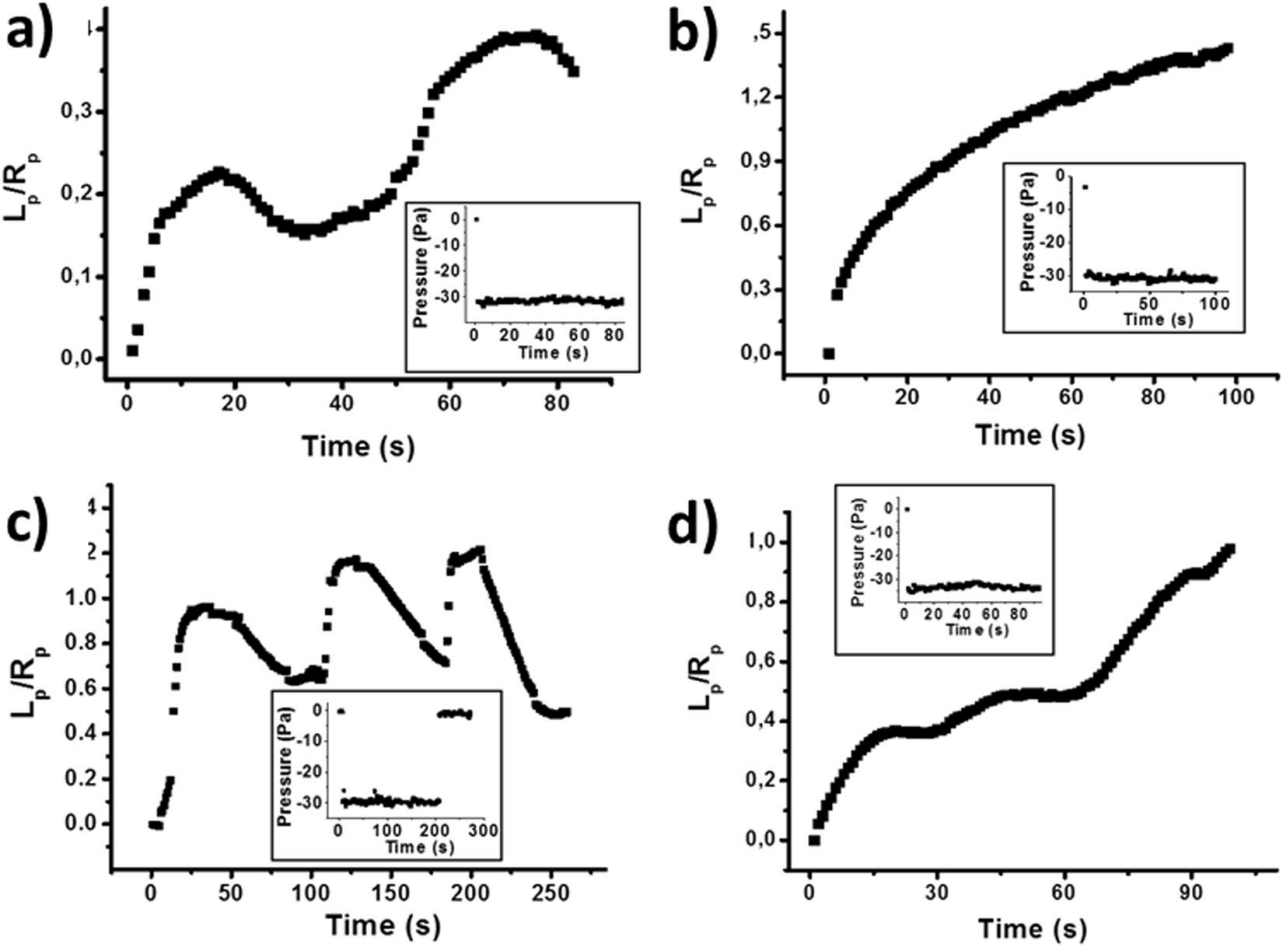
Most commonly found behaviors for the cell protrusion once K562 cells are exposed to OTC and they are subjected to a fast pressure jump. (**a**) oscillating behavior with no evidence of bleb formation; (**b**) continuously growing cell projection which can be fitted by a power-law relationship; (**c**) oscillating behavior with the formation of membrane blebs causing the rapid progression of the cell protrusion followed by a retraction; (**d**) alternating phases of stability followed by positive increases of the cell projection. The inset in each image represents the corresponding values of the applied pressure during cell creep analysis.

**Figure 3:**
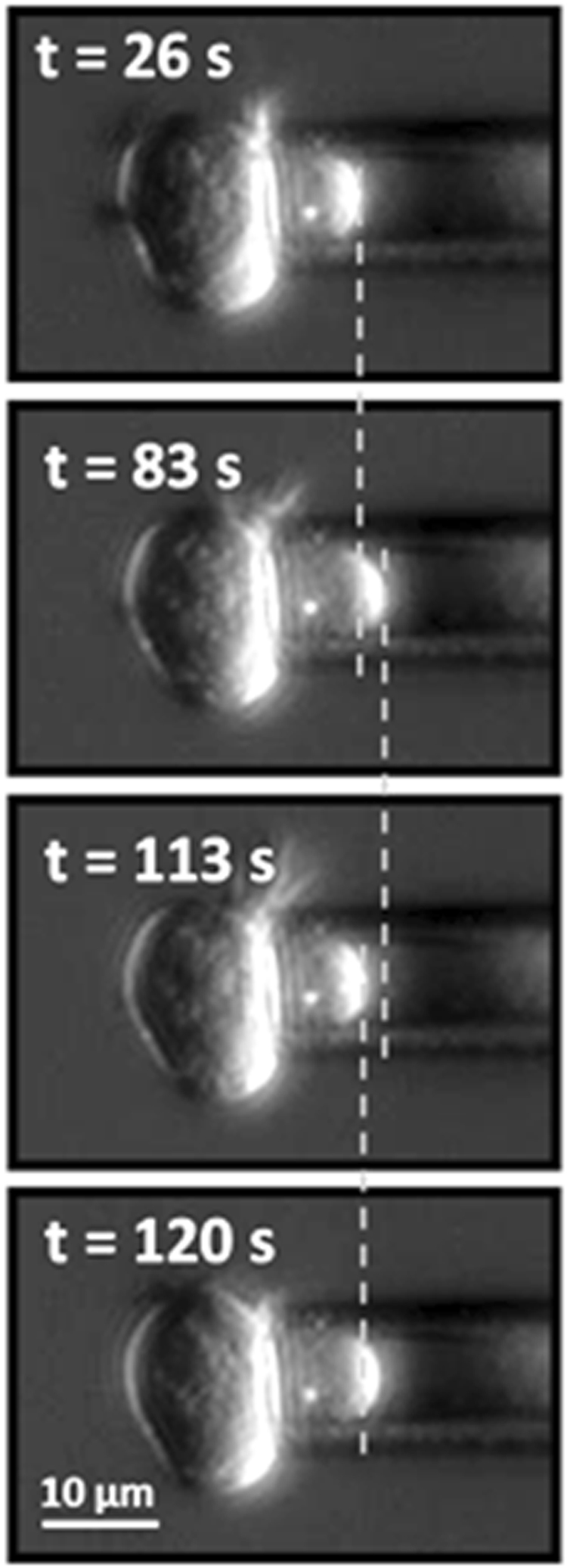
Snapshots representing the protrusion of a cell undergoing an oscillatory behavior. At time t = 0 s a negative pressure step was applied to the cell. Each image shows the corresponding acquisition time after the pressure step. The vertical dashed lines have been inserted to immediately get the cell protrusion position with respect to the previous snapshot.

We then concentrated on K562 cells exposed to OTC for different incubation times. We noted that by increasing the incubation time of OTC the cells started to present other specific creep behaviors with an increase of the cases ascribable to high cellular cortical activity. We classified the main behaviors in four different categories and they are represented in Fig. 2: a) growth of the protrusion followed by retraction and a new growth with no evidence of bleb formation (see Fig. 3 for an example); b) continuous growth of the cell protrusion with a progression that could be fitted by the power-law model in a Log-Log plot with a correlation coefficient higher than 0.99; c) oscillating behavior characterized by alternating growth and retraction cycles with a very rapid growth step and evident formation of large cell blebs (see Figure S6 for this particular case); d) step-growth characterized by growth followed by stabilization and then a new growth and stabilization cycle (saltatory behavior). This last case is distinguished with respect to the case reported in Fig. 2b by the fact that a linear fit in a Log-Log plot results in a correlation coefficient with a feature of 0.98 or lower. Representative movies for different behaviors are reported in the Supplementary Information (Movies S1–5). Another type of behavior that we already mentioned is the almost immediate retraction of the cell protrusion after the application of the pressure step. We also performed a detailed analysis of the behavior at short time in the cases where the oscillatory behavior at longer time was found. The analysis (see Fig. S7) shows that the power-law is a good model to describe the short time behavior of the cell protrusion and the value of the α exponent that we found (we didn’t perform a statistical analysis for the different incubation times) was in all cases higher than the value obtained for cells showing a power-law behavior on a longer time scale.

**Figure 4:**
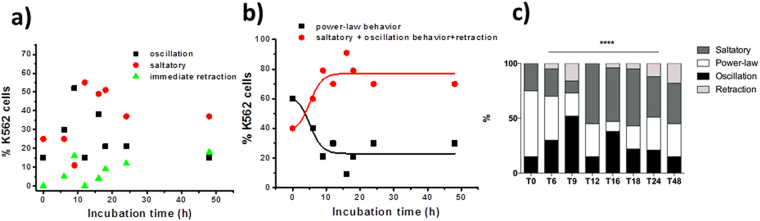
(**a**) Variation of the percentage of cells following the different behaviors as a function of the incubation time in OTC; (**b**) Sigmoidal fits to the data representing the percentage of cells undergoing the power-law behavior and percentage of cells undergoing the oscillatory, saltatory or immediate retraction behavior as a function of the incubation time; (**c**) histograms showing percentage of cells exhibiting the different behaviors reported in Table 1 for the different incubation times in OTC (control – no OTC incubation; T6-6 h; T9-9 h; T12-12 h; T16-16 h; T18-18 h, T24-24 h, T48-48 h) (****p < 0.0001). Data were analyzed using GraphPad Prism 6 software (GraphPad Software, Inc., La Jolla, CA, USA). The Chi-squared test was used for cell protrusion behaviors. *p < 0.05 was considered significant. A significant difference among the different cell treatments (incubation times in OTC) was observed with respect to the control (no incubation with OTC).

We found that the percentage of cells following different behaviors changed in a way correlated with the OTC incubation time. Table S1 in the Supplemental Material reports the percentage of the different cases for the different incubation times we concentrated on whereas Fig. 4a shows the trends of the single types of behavior together with a statistical analysis to determine the presence of significant differences between the different incubation times and the control experiment (Fig. 4c). Considering that the trends of the single behaviors reported in Fig. 4a are not able to show a clear trend, in Fig. 4b we reported the comparison between the number of cells undergoing the power-law behavior and the number of cells showing behaviors ascribable to active mechanics (oscillatory, saltatory or immediate retraction) as a function of the incubation time. We also included a sigmoidal fit to the two groups of data. It is evident that the percentage of cells whose behavior could be described by a power-law relaxation process decreases as the OTC incubation time increases whereas the occurrence frequency for behaviors ascribable to increased contraction of the acto/myosin complex rises.

**Figure 5:**
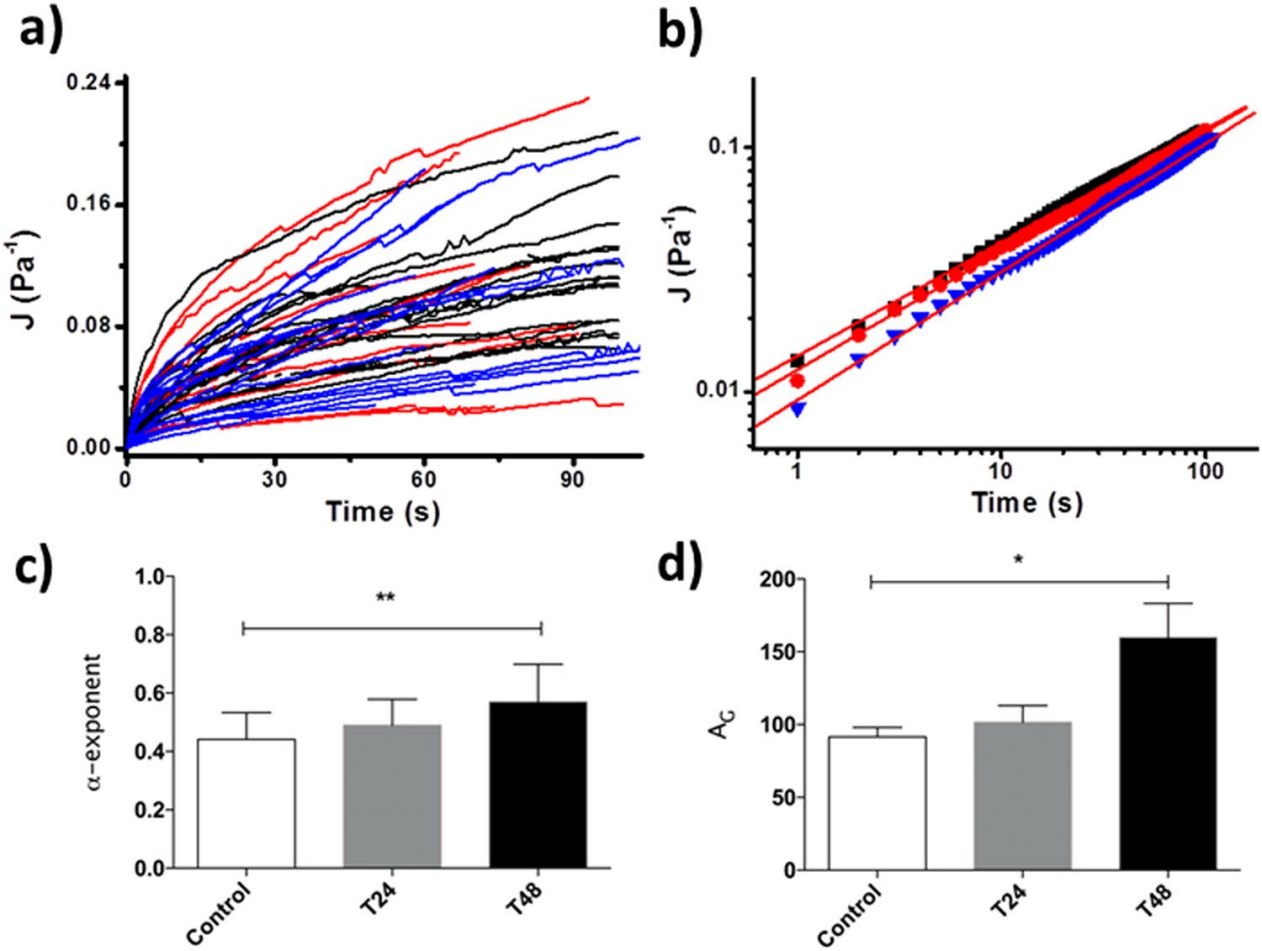
(**a**) Plot of the cell protrusion creep compliances as a function of the incubation time in OTC: control (untreated cells) (black curves), 24 h (red curves), 48 h (blue curves); (**b**) averaged curves of the Log-Log plots of the behaviors in **a**). The red continuous lines are the fit with the power-law relation. (**c**) Statistical analysis of the value of the α- exponent corresponding to different incubation times in OTC (control, T24-24 h; T48-48 h) (mean ± SEM). In the case of control vs T48 we obtain **p < 0.01 (Kruskal-Wallis test). (**d**) Statistical analysis of the value of the stiffness parameter corresponding to different incubation times in OTC (control, T24-24 h; T48-48 h) (mean ± SEM). In the case of control vs T48 we obtain *p < 0.1 (Kruskal-Wallis test).

In cases of cells showing a continuously growing protrusion inside the micropipette (well described by a power law function) even after the exposition to OTC, we extracted an average behavior and we obtained the values for the power-law exponent and for the stiffness parameter. We concentrated on the incubation times of 24 h and 48 h compared to the control case. As shown in Fig. 5, cells presented an increase of the fluidity upon an increase of the incubation time with OTC and also an increase of the stiffness parameter. The numerical values of the two different parameters for the different incubation times have been reported in Table S2. The differences we obtained for the α exponent and the stiffness parameter between the control cells and the cells incubated in OTC for 48 h are statistically significant, In Figure S8 we reported the distributions of the α and stiffness parameters for control cells, cells incubated for 24 h and 48 h with OTC. At the same time, in Figure S9a we analyzed the correlation between the fluidity of cells and their stiffness parameter, We found that an increase of the fluidity is typically associated with an increase of the cell stiffness. We also established that in the condition used for the experiments there is no correlation between the radius of the cells and the obtained fluidity parameter (Figure S9b).

**Figure 6:**
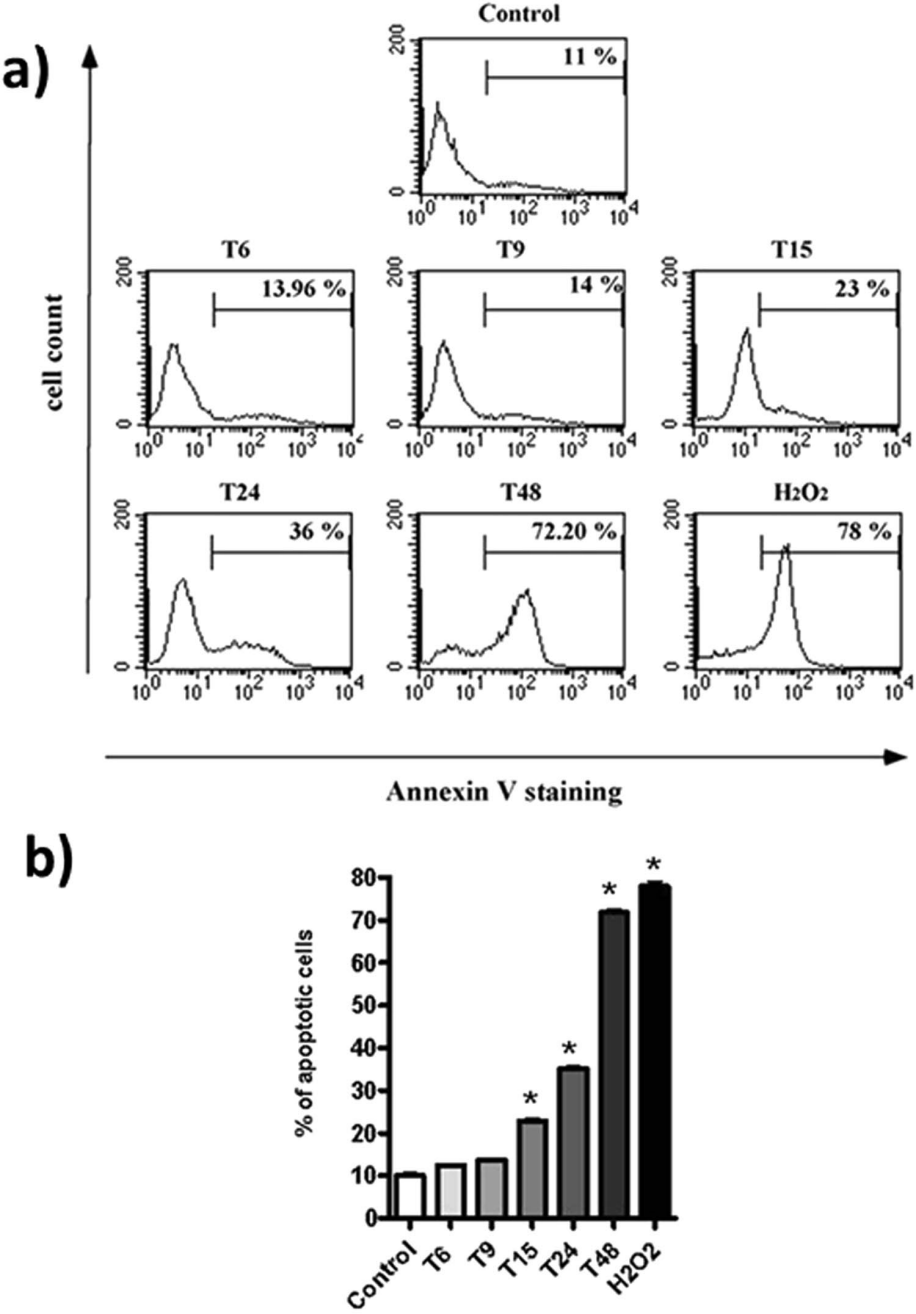
Panel a refers the Apoptosis induction evaluated as fluorescence intensity of fluorescein isothiocyanate–Annexin V-staining in one representative experiment. The histogram plots show the staining peaks in the different conditions for the K562 cell line cultures (Control = untreated cells; T6-T48 = OTC incubation for 6 h, 9 h, 15 h, 24 h, 48 h, respectively; H_2_O_2_ = represents the positive control of apoptosis induction on K562(see Materials and methods). The percentages of the K562 cells labeled with Annexin V are reported in each histogram plots. The amount of the apoptosis induction is related to the shifting to the right of the peak on the x-axis; Panel b refers the bar graph data (mean (percentage) ± SD) of the 3 performed experiments based on Annexin V staining for apoptosis evaluation. Control represents the untreated cells and refers to the apoptosis background (growth medium without OTC); T6-T48 represent the apoptosis evaluation at the different incubation times of the K562 cell line in a growth medium with the addition of OTC. As positive control of apoptosis induction is reported the effect of H2O2 (see Materials and methods). Thestatistical significance of each incubation time vs the negative control (untreated cells) is indicated with asterisk (P ≤ 0.005, with Paired T Test).

We previously reported that OTC causes the induction of apoptosis in human cell line K562 and in human peripheral blood cells (PBMC)^36,46^. Here, we investigated the cytotoxic effect of OTC after incubation at shorter time-scales (see the Materials and Methods section). As a marker for the apoptotic process we used the translocation of phospatidylserine to the external leaflet of the membrane exploited by the cells to increase the recognition ability by macrophages. The results are reported in Fig. 6. Here, we confirmed the toxicity of OTC in terms of apoptosis induction^36^. Notably, the pro-apoptotic effect of OTC seems to be stringently time-dependent: it was evident from T15, clearly observed at T24 and frankly occurred at T48, as previously described^36^. In contrast, the short incubation times with the drug (6 h and 9 h) provide a percentage of apoptotic cells very similar to the untreated cells, used as negative control. In this regard, the Annexin staining on untreated cells represents the physiological probability of naturally occurring apoptosis during K562 cell line culture.

**Figure 7:**
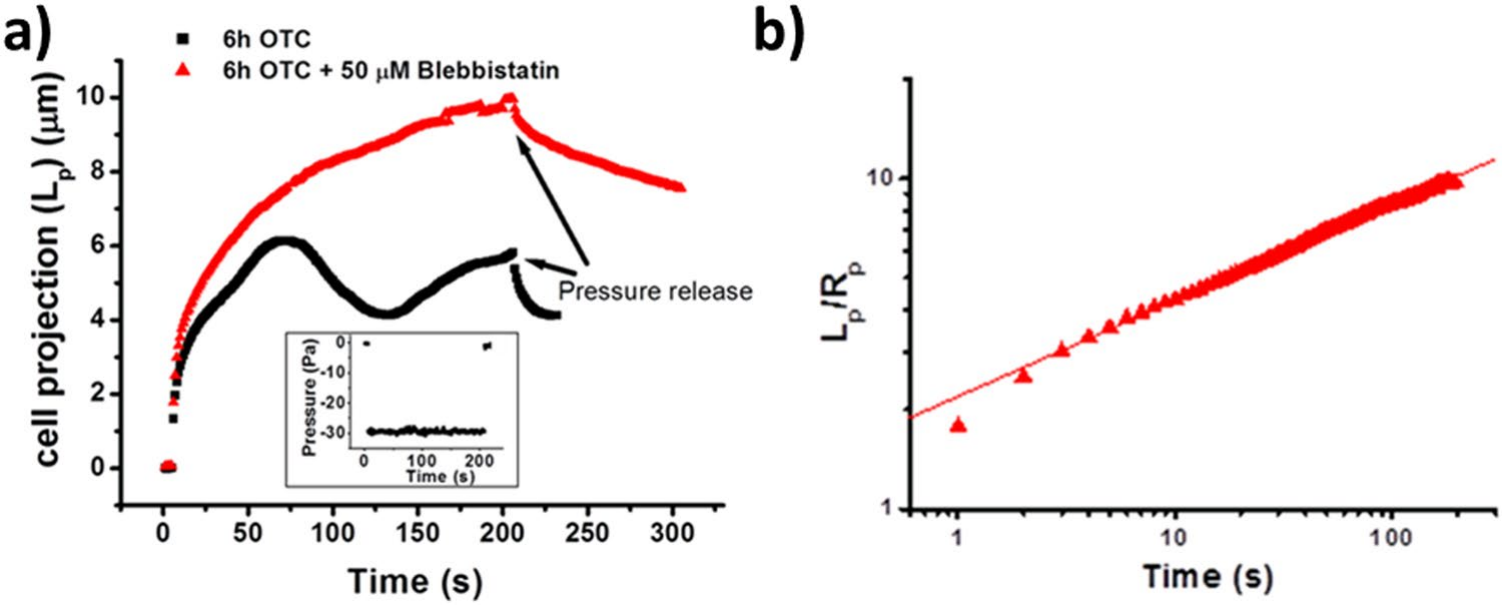
Effect of blebbistatin on K562 cell exposed to OTC and showing an oscillation behavior. (**a**) The black squares represent the behavior of the protrusion of a cell exposed for 6 h to OTC. After 200 s the pressure jump is removed and the pressure is taken back to the initial value. The red triangles represent the behavior of the same cell after it has been exposed to 50 μM blebbistatin for 10 min; The inset reports the corresponding values of the pressure drop during the creep analysis before blebbistatin injection; (**b**) Log-Log plot of the cell in (**a**) after it has been exposed to blebbistatin.

To shed further light on the oscillating and retraction behavior of cells inside the micropipette at constant applied pressure, we raised the hypothesis that this behavior was due to the contractile acto/myosin II complex and we studied the possibility of removing the cell retraction behavior by using blebbistatin, a well-established inhibitor of myosin II activity. Blebbistatin is known to leave myosin II in a configuration in which its head is not able to bind to actin in order to exert its contractile activity^51,52^. When we found cells showing oscillating behavior, we repeated the pressure jump protocol to be sure that the oscillations were not present just in the first run (Figure S13). Then we injected blebbistatin in the chamber to reach a final concentration of 50 μM while applying the lowest possible pressure (less than about 9 Pa), which assured keeping the cell in contact with the micropipette. After 10 minutes to allow blebbistatin reaching a uniform concentration in the cell chamber, we applied the pressure jump again. Figure 7 shows that after exposing the cell to blebbistatin, the oscillating behavior is removed in favor of a continuous growth of the protrusion. Moreover, the creep behavior in the presence of blebbistatin is well fitted by the power-law model with an exponent value of 0.29 ± 0.08 (mean ± SD on the basis of three experiments – see Fig. S14). We also considered the possibility that incubating the cells with OTC in the presence of blebbistatin could prevent the effect of OTC on the active behavior of the cells. Cells were incubated for 12 h in the presence of both drugs. This experiments allowed also verifying that 50 µM blebbistatin is not toxic for our cells. After the incubation, blebbistatin and OTC were removed from the medium and the cells were analyzed by MAT. Even in this case, cells showed an increase in the frequency of the oscillatory behavior (see Fig. S15) highlighting that blocking myosin is not effective in preventing the effect of OTC.

## **Dis**c**ussion**

There are well-established relations between the apoptotic process and the cytoskeleton, especially the actin network^53,54^. Different apoptotic stages are characterized by large actin polymeric network reorganization. The most evident structural reorganization is the increase of the cellular cortical tension due to the acto/myosin II contractile system in the first stages of apoptosis. Myosin II light chain is increasingly phosphorylated leading to an increased contractile activity^55^. This reorganization leads to the detachment of the plasma membrane from the cortical cytoskeleton and the formation of membrane blebs. This process is controlled by the activity of Rho GTPases or the Rho effector ROCK1^56^. Apart from the initial stages of the apoptotic process, other cytoskeletal processes, such as actin depolymerization, play a fundamental role for the last stage of apoptosis. Many drugs that are active on the actin cytoskeleton have been demonstrated to induce apoptotic processes on specific cell types^54^ with a relevant role played by the altered cytoskeletal actin dynamics. Caspases are known to be also active on proteins such as Actin Binding Proteins (ABP), which establish connections between the cortical cytoskeletal layer and the membrane. If these connections are removed, blebbing of the plasma membrane gets much more probable in the presence of small pressure differences between the inside and outside of the cell.

According to the strong relation between the apoptotic process and the cytoskeleton structure, it is reasonable to expect specific mechanical signatures in cells undergoing apoptosis. For example, Lam *et al*.^57^ demonstrated that the first stages of apoptosis for suspended cells are characterized by a marked increase of the cell stiffness. In the case of adherent cells the relation between cell mechanical properties and apoptosis has been studied using AFM by Pelling *et al*.^58^. The authors found that apoptosis induced by staurosporine is connected to an organized cell mechanical alteration characterized by a non-monotonic trend. After an initial rapid decrease of the cell Young modulus, an increase is observed again followed by a continuous decrease.

In the literature, cases in which cells kept at constant applied stress show a reaction corresponding to active behavior have already been reported. Using a microfluidic optical stretcher, Chan *et al*.^59^ showed that in the case of HL60 cells, a temperature higher than 52 °C induces an active retraction of the cells after the initial deformation. The initial temperature increase induced an increase of cellular fluidity but above 52 °C, a trend inversion was observed at longer time-scales. This behavior has been explained by the increased activity of temperature-sensitive Ca^2+^ channels. Intracellular calcium is then able to increase the activity of myosin II introducing an active behavior of the cells.

When a cell is aspirated inside a micropipette, the state of the acto/myosin cortical layer plays an important role in establishing the way in which the cell react and the activity of myosin II is fundamental to resist the deformation. Typically, the cortical layer has a thickness spanning from 100 nm to 1 μm and the contractility due to myosin II is due to different aspects: its abundance in the layer, the binding affinity with actin and its phosphorylation state. The cortical tension is a combination of viscoelastic contributions due to the dynamic activity, polymerization and depolymerization, of the cortical actin, and contributions due to the contractile activity of myosin II. It has been found that the myosin II/actin bond can be modeled as a catch-bond, implying an increase of the affinity when a force trying to break the bond increases^34^. An increase of the force applied to the bond could be the result of a dilation strain in the cell cap inside the micropipette. In this context, by modeling cell deformation in a micropipette subjected to a negative pressure it has been established that the cap region is the area where the dilation strain is concentrated^34^. The increased affinity of myosin II in this particular cell region induces a local accumulation of the molecular motor and this could increase the cortical tension leading to a retrograde movement of the cell protrusion at constant applied pressure. Once the strain decreases, the affinity of myosin for actin could decrease again, inducing a reduction of its accumulation in the cap region^34^. As a consequence, the protrusion will start to increase again and an oscillatory behavior could be established. Apart from this explanation for the oscillatory behavior, another mechanism is possible. In fact, it is possible that the increased cortical tension could induce the detachment of the plasma membrane from the cortical actin with the formation of blebs. The higher the activity of myosin II is, the higher the probability that this event occurs. In fact, we observed an increased probability of cell blebbing when cells have been exposed to OTC compared to the control. Once a bleb is formed, the cell protrusion inside the micropipette undergoes a fast inward movement and the membrane is no more able to transmit the force to the cortical actin. What happens after a plasma membrane bleb has formed has been thoroughly studied^60^. The presence on the plasma membrane of nucleation sites for the actin cytoskeleton induces, immediately after a bleb is formed, actin polymerization underneath the membrane and the rapidly growing actin network produces a contraction of the membrane bleb, taking it back to the rest of the cortex. The continuously growing actin network can once again induce an increase of the cortical tension reaching the threshold where another bleb starts. By this mechanism, an oscillatory behavior due to bleb formation could be produced. We think that the difference between the two cyclic behaviors is marked by the kinetics of the cell protrusion increase. Indeed, in the case of large bleb formation the protrusion increase is very fast and the plot showing the tracking of the protrusion inside the micropipette forms a sort of cuspid in the lower portions (see Fig. 2c) whereas, without plasma membrane detachment the plot is smoother (see Fig. 2a).

**Figure 8:**
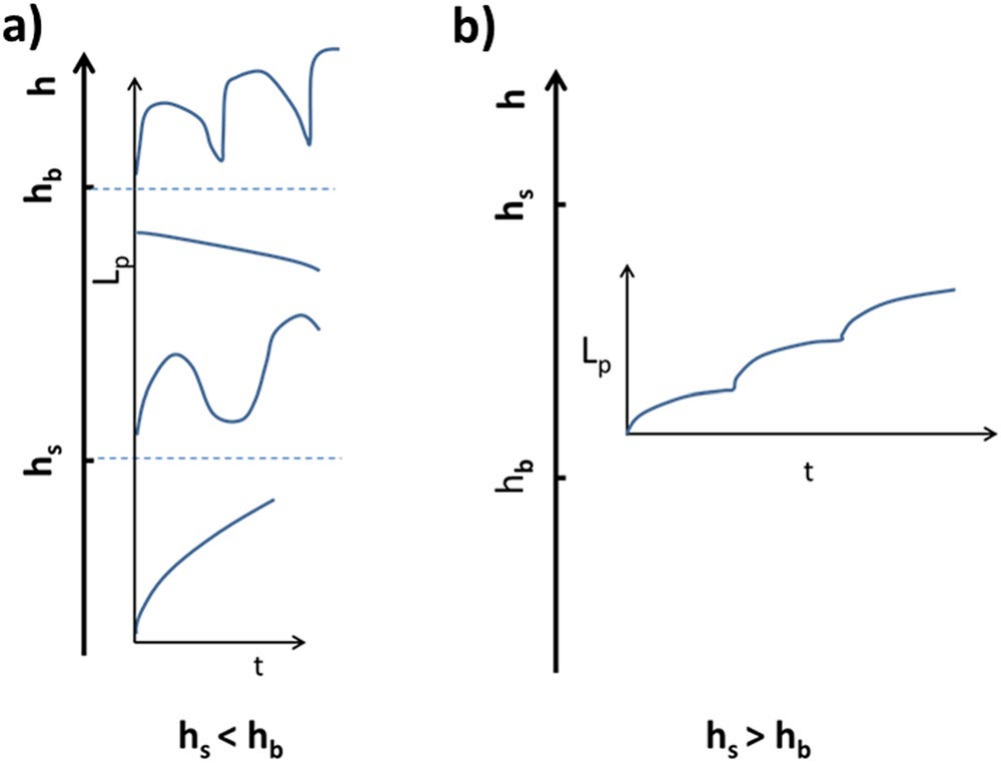
Phase diagram for the possible behaviors of the cell protrusion inside the micropipette as a function of the thickness of the cortical layer, *h*, with respect to the critical thicknesses. The different lines represent the trend of the cell projection *L*_*p*_ as a function of time, t. (**a**) Case in which the threshold thickness for protrusion retraction, *h*_*s*_, is lower than the thickness threshold for plasma membrane detachment from the actin cortex, *h*_*b*_. From the top: cell oscillations with large bleb formation, instantaneous retraction; oscillations with no bleb formation, continuous growth. (**b**) Case in which *h*_*s*_ is bigger than *h*_*b*_. Saltatory behavior: tongue increases to a stationary situation followed by a new increase

In a very interesting paper Brugués *et al*.^61^ developed a model to explain the different behaviors which can be observed when cells are exposed to a negative pressure jump inside a micropipette. Starting from the equilibrium condition between the cortical tension and the applied pressure given by^61^:

4 $${\sigma }_{myo}h={\rm{\Delta }}P{R}_{P}/2$$

where σ_myo_ is the contractile stress of myosin and *h* is the cortical thickness (the product on the left side represents the cortical tension). If the equilibrium situation is not satisfied there will be a movement of the cell protrusion (see Supplementary Material). In particular, the increase of the first member of Equation 4 could produce a retraction of the cell tongue. Here we neglected the contribution coming from the plasma membrane tension (from the lipids), which is negligible with respect to the cortical one. This condition determines a threshold value for the cortical thickness, *h*_*s*_, above which a retraction behavior is produced. In ref.^61^ it has also been considered that there is a limit value for the force, ${f}_{b}^{\ast }$, the bond between the cortical actin and the plasma membrane can sustain before a bleb is formed. This threshold limit depends on the state of the molecules assuring this kind of binding and once again on the myosin activity. The threshold force is connected to another thickness threshold for the cortical actin, *h*_*b*_, over which blebs are formed (cortex detaches). The two limits are given by the following expressions^61^:

5 $${h}_{s}=\frac{{\rm{\Delta }}P{R}_{P}/2}{{\sigma }_{myo}}$$

6 $${h}_{b}=\frac{{f}_{b}^{\ast }{R}_{P}/2}{{\sigma }_{myo}{\xi }^{2}}$$

where ξ represents the mesh size for the cortical actin. Depending on the myosin activity these two threshold values might change and they can affect the behavior of the cell protrusion. Brugués *et al*. obtained a sort of phase diagram representing these different situations. A phase diagram slightly modified with respect to the one reported in Brugues *et al*. is reproduced in Fig. 8^61^. We modified the phase diagram by inserting a state in which an oscillation behavior is present also in the presence of cytoskeletal attachment to the plasma. The driving force for these oscillations comes from the strain dependent actin/myosin II affinity as explained above^34^. Among the different possible behaviors there is the continuous growth of the projection length which occurs when the cortical thickness doesn’t exceed the critical thicknesses together with the condition *h*_*b*_ > *h*_*s*_. A low value of the myosin activity could increase *h*_*s*_ and *h*_*b*_ values assuring a cell cortical thickness value lower than *h*_*s*_. This is the situation we mainly observed for control K562 cells. In these cases we found that a power-law relation can describe the cell mechanics. The same behavior has already been found for the same type of cells^26^. The feature for the exponent we obtain is 0.44 ± 0.02 whereas the cell stiffness at *t*_0_ = 1 s is 91 ± 6 Pa. Other behaviors are observed for control cells, but in a limited number of cases (see Table S1) that could represent the normal percentage of apoptotic cells in an untreated population. The main result of the investigation of the cells, which have been exposed to OTC, is that other behaviors for the cell protrusion inside the micropipette appear and the percentages of cells following each behavior change with the incubation time in OTC. In particular, when increasing the incubation time in OTC, a higher number of cells is subjected to an oscillating behavior. This trend could be explained on the basis of a strong increase of the myosin II activity that could be associated with the rising of the apoptotic process. Starting from 6 hours of OTC incubation, a significant variation with respect to the control condition is found. When increasing the incubation time, the saltatory behavior seems to become the prevalent one. In this case *h*_*s*_ is larger than *h*_*b*_. This condition could be obtained in different ways. One possibility is to increase the aspiration pressure. Another possibility is associated to the decrease of the limit force before the detachment of the plasma membrane from the actin cortex ${f}_{b}^{\ast }$. Considering that the experiments have been performed using the same pressure-jump value, we speculate that the apoptotic process could affect the proteins, which are involved in keeping the actin cortex anchored to the plasma membrane. It has been shown that ERM (Ezrin/Radixin/Moesin) proteins, which are responsible for the actin-cortex/plasma membrane attachment, migrate from the plasma membrane to the cytoplasm during the first stages of the apoptotic process^62^.

In the case of OTC exposure, a variable fraction of cells still continued to show a power-law behavior. For specific incubation times (control, 24 h and 48 h) we averaged all the curves recorded on cells showing this behavior. We noticed (Fig. 5) that the cells become more fluid when exposed to OTC (the exponent α increases). This behavior could suggest augmented fluidity as a consequence of an increased activity of myosin II. Table S2 reports the analysis of the curves for the different incubation times giving the values for the fluidity and the cell stiffness parameters. Together with an increase of the α value we observed an increase of the stiffness parameter measured at 1 s. Considering the relationship between the α exponent and the stiffness, different results are present in the literature. Experimental approaches based on Micropipette Aspiration showed that the increase of the fluidity parameter is typically associated to an increase of the stiffness parameter (see for example: ref.^25^). It is like a cell becoming stiffer at short time is also more able to flow. This is the same trend that we observed in our case. Using other techniques such as microfluidic approaches where cells are induced to pass through microconstrictions by the application of high pressure, pieces of evidence have been found concerning the presence of a master equation connecting an increase of the fluidity parameter to a decrease of cell stiffness (see for example: ref.^26^). This behavior is opposite to the one we found in our case. We think that the difference in the two behaviors could be connected to the fact that the two techniques consider very different time scales for the cell deformation (seconds in the case of micropipette aspiration and milliseconds in the case of microfluidic approaches).

It has already been proposed that the contractile activity of myosin II could help disentangling an actin network^63^ especially at longer time-scale (a few seconds). Accordingly, an augmented myosin II activity, as expected in the very first stages of the apoptotic process, could be the reason for the increase of the cell fluidity, similarly to the increased fluidity observed for high temperature. Another possible reason for the increased fluidity when myosin II activity is enhanced is connected to the depolymerization of actin filaments^64^. The difference between the two possibilities, disentangling activity and depolymerization process, could be related to the time-scale of the effect. If the effect is observed at short time-scales (in the milliseconds range) myosin II could not have enough time to induce a disentanglement of the actin network. If the effects are present only for longer time-scales (in the range of seconds) the disentanglement phenomenon could be the prevailing one. According to this consideration we think that the increased activity of myosin II in the very initial stage of apoptosis could produce a disentanglement effect on the actin network resulting in enlarged cell fluidity.

### Conclusions

We showed that a drug (Ossitetraciclina liquida 20% - OTC), which is known to induce apoptosis in a specific cell model *in-vitro*, is also able to affect the mechanical phenotype of the same cells. By using the Micropipette Aspiration Technique, we found that the cells become more fluid when exposed for increased time-intervals to OTC and the probability of an active behavior was enhanced, manifested by a retrograde movement of the cell tongue inside the micropipette while aspirated by a constant pressure difference. Cell behavior analyzed by MAT changed significantly with respect to control cells after an incubation time of just 6 h, whereas, by using the Annexin V marker, a variation from the control cells is observed for incubation times of 15 h or longer. All the mechanical phenomena we observed could be traced back to an increased activity of the myosin II molecular motor. Considering that in the first stages of the apoptotic process myosin II activity is increased we proposed that what we observed is due to the initial phases of the apoptotic process. The obtained results also suggest that the mechanical phenotyping of living cells could be exploited to detect the early stages of pathological processes.

## Electronic supplementary material

supplementary material

Movie S1

Movie S2

Movie S3a

Movie S3b

Movie S4

Movie S5
