# Supplementary material for: Mechanical phenotyping of K562 cells by the Micropipette Aspiration Technique allows identifying mechanical changes induced by drugs
Source: Sci Rep. 2018 Jan 19;8:1219. doi: 10.1038/s41598-018-19563-z (PMC5775209; doi:10.1038/s41598-018-19563-z)
Supplement: Supplementary file 1 — supplementary material [file 41598_2018_19563_MOESM1_ESM.pdf]

Supplementary Material for:

Mechanical phenotyping of K562 cells by the Micropipette Aspiration Technique allows identifying mechanical changes induced by drugs

Alessandro Di Cerbo[a,§], Valentina Rubino[b,§], Francesca Morelli[a], Giuseppina Ruggiero[g], Rosaria Landi[h], Gianandrea Guidetti[c], Sergio Canello[d], Giuseppe Terrazzano[b,e], Andrea Alessandrini[a,f,*]

[a] Department of Physics, Informatics and Mathematics, University of Modena and Reggio Emilia, Via G. Campi 213/A, 41125 Modena, Italy;

[b] Department of Molecular Medicine and Medical Biotechnology, University of Naples Federico II, Via Pansini 5, 80131 Naples, Italy;

[c] Division of Research and Development, SANY*pet* SpA, Via Austria 3, 35023 Bagnoli di Sopra (PD), Italy;

[d] Research and Development Department, Forza10 USA Corp, ORLANDO FL, USA

[e] Department of Science, University of Basilicata, Via Sauro 85, 85100 Potenza, Italy;

[f] CNR-Nanoscience Institute- S3, Via Campi 213/A, 41125 Modena, Italy

[g] Department of Translational Medical Sciences, University of Naples Federico II, Via Pansini 16, 80131 Naples, Italy

[h] Department of Molecular Medicine and Medical Biotechnology, University of Naples, Federico II, Via Pansini 5, 80131 Naples, Italy

[§] These authors contributed equally to the work

e-mail. andrea.alessandrini@unimore.it, Alessandro811@hotmail.it,

Supplementary Material and Methods section

Basic theory for micropipette aspiration

The equilibrium condition for the aspiration inside the micropipette is given (considering Laplace law) by:

$$\Delta P = 2\left(\frac{\gamma_{in}}{R_p} - \frac{\gamma_{out}}{R}\right) \quad \text{Eq. S1}$$

where ΔP is the applied pressure difference, R_p is the internal micropipette radius, R is the radius of the cell outside the micropipette and γ_{in} and γ_{out} are the overall cortical tensions for the region inside and outside the micropipette, respectively. If the equilibrium condition is not satisfied there will be a motion of the cell projection inside the micropipette. Typically, R is much higher than R_p and the second term inside the parenthesis is negligible with respect to the first one. The cortical tension is given by the product of the contractility due to myosin (σ_{myo}) and the thickness of the cortical region h: σ_{myo} h. ΔP can be considered the driving force for the movement of the cell protrusion whereas 2 γ_{in}/R_p can be considered the restoring force. If the two terms are not in equilibrium there will be a movement of the cell protrusion. The dissipative term during the strain relaxation is due to the reorganization of the cytoskeletal components and the viscosity of the cytoplasm.

Image analysis

The automatic detection of the cell protrusion is based on the following protocol:

a) The images of the sequence are initially 2D aligned using a feature present in each image and which should be always in the same position (typically a detail of the micropipette)
b) The very last part of the protrusion is then used to perform a new 2D alignment using this feature as the template. The recognition of this feature in each image leads to a lateral displacement of each image with respect to the initial reference. The lateral displacement of each image is reported by a data list in ImageJ. The value of the lateral displacement can be exploited to obtain the variations of the cell protrusion length inside the micropipette.
c) Each translated image is then visually checked to look for possible wrong recognition events. In this case the procedure is repeated by slightly changing the selected template.
d) In the analysis the very last part of the cell protrusion is always selected. It means that, in the case of the formation of blebs, the position of the membrane bleb is measured.

To quantify the effect of the initial value of the pressure used to keep the cell in contact with the micropipette on the overall creep compliance behavior we took some points (about 200 s) before the application of the fast negative pressure step. Figure S1 shows a typical experiment where it is evident that the cell protrusion movement is negligible in the first 200 seconds with respect to the movement induced by the fast pressure step.

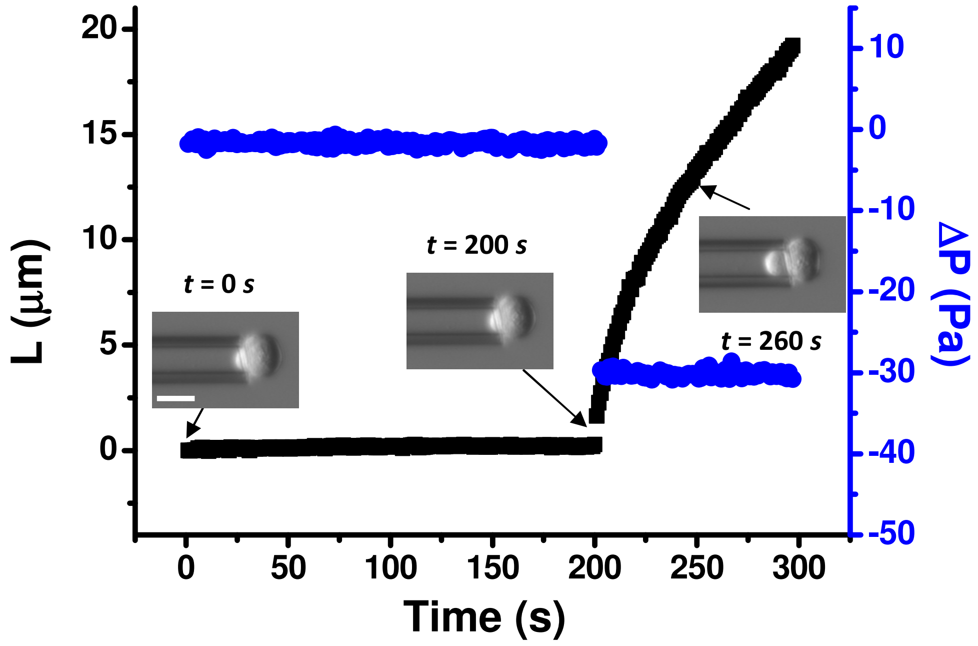

Figure S1: Analysis of the cell protrusion movement (black points – the initial position is taken as the zero-position value) in the case of a pressure protocol represented by the blue points. The experiment has been performed to verify that the initial pressure value doesn't affect the cell protrusion position even for long holding time intervals. The bar in the inset is 10 μm.

Imaging conditions

All the experiments were performed in ambient gas and at room temperature. We typically changed the chamber containing the cells we were working on every hour taking new cells from the incubator. We relied on the color of the medium to try to maintain the pH of the solution as much as possible constant. We found that also in the control sample some cells showed a behavior typical of dead cells (they cannot be sucked inside the micropipette with pressure drops up to 500 Pa). The probability of finding these types of cells was not greatly affected by the incubation time with OTC.

We also constructed a set-up in which we were able to control the temperature of the cell chamber. To work at high temperature (37°C) we also had to implement a system to increase the humidity (exploiting an ultrasonic humidifier – see scheme and pictures in Figure S2) in order to slow down the evaporation of the medium. However, due to the constraint of an open set-up to allow the manipulation and the entry of the micropipette in the chamber we were not able to reach a relative humidity higher than 70%. This situation allowed us to work only for about 15 minutes before evaporation was evident in the chamber. Anyway, considering experiment of the same cell at temperatures from 30 to 37 °C we observed mainly a quantitative difference in the behavior after the same pressure drop but the type of cell protrusion behavior was similar (Figure S3). At higher temperature the cell deformation was larger. Considering that our analysis was mainly a comparison

between cell exposed to OTC for different incubation times but measured in the same conditions we consider our results representative of the effect of OTC.

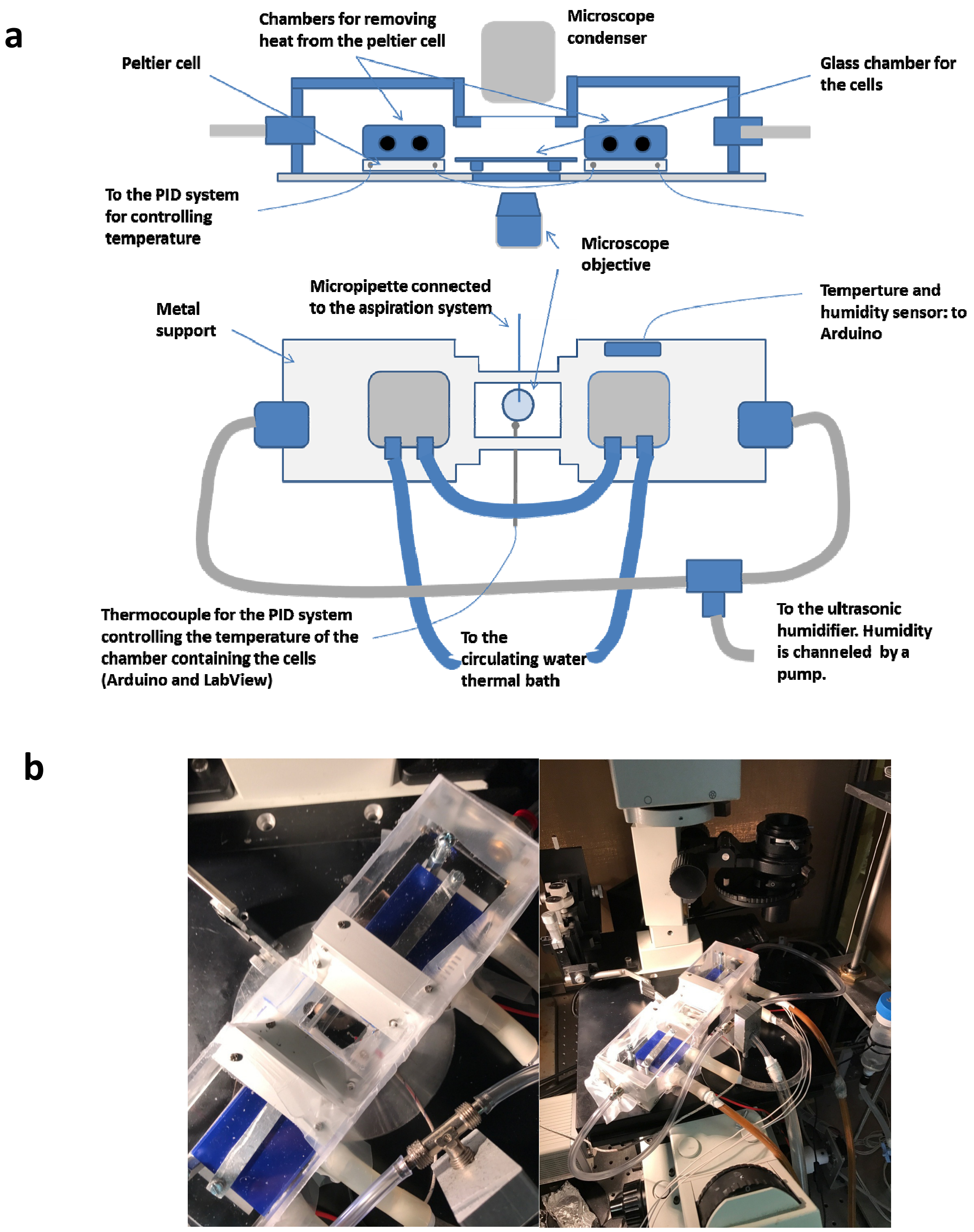

Figure S2: a) Schematics of the set-up developed to test differences in the behavior of the cell protrusion for different temperature conditions. In the chamber containing the cells the temperature is controlled by a PID circuit and the humidity of the environment is increased by exploiting an ultrasonic humidifier. b) pictures of the set-up installed on the optical microscope stage.

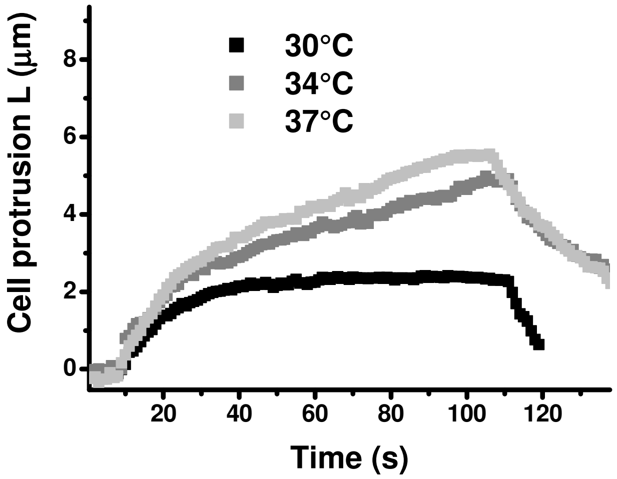

Figure S3: Example of a cell exposed to the same negative pressure step at 3 different temperature values. Increasing the temperature the cell appears softer but the qualitative behavior is always the same.

Equivalence between the frequency and the time relaxation analysis

Rheological characterizations of living cells by using different techniques such as MCT and AFM demonstrated that the soft-glassy model can be used to describe their mechanical behavior. A typical exploited protocol is based on the application of a sinusoidal stimulus to the cell while the deformation and the phase lag between the applied stimulus and the cell deformation are measured. By using this approach, both the elastic and the dissipative behaviors are obtained. The soft-glassy model is translated into a power-law analytical relation between the mechanical parameters and the frequency of the applied stimulus according to the following expression:

$$G^*(\omega) = A_G (i\omega)^{\alpha} = G'(\omega) + iG''(\omega) \qquad \text{Eq. 1}$$

where A_G is the shear stiffness constant and $\omega = 2 \pi f$, where f is the frequency of the applied stimulus. In the case of the time domain, the power-law model foresees a dependence of the shear creep compliance on time according to the following equation:

$$J = A_J t^{\alpha} \qquad \text{Eq. 2}$$

In the previous expression A_J represents the value of J for t = 1s. The two exponents in Eqs. 1 and 2 have the same value and it can be demonstrated that:

$$A_G (i\omega)^{\alpha} = \frac{1}{A_J \Gamma(1+\alpha)} (i\omega)^{\alpha} \qquad \text{Eq. 3}$$

where Γ (..) is the gamma function. According to the previous equations, the stiffness parameter in Pa can be obtained from the fit of the power-law expression to the relaxation trend [1]:

$$A_G = \frac{1}{A_J \Gamma(1+\alpha)} \qquad \text{Eq. 4}$$

[1] Zhou, E. H., Quek, S. T. & Lim, C. T. Power-law rheology analysis of cells undergoing micropipette aspiration. Biomech Model Mechanobiol 9, 563-572, (2010)

Comparison between the power-law model and the four elements' model

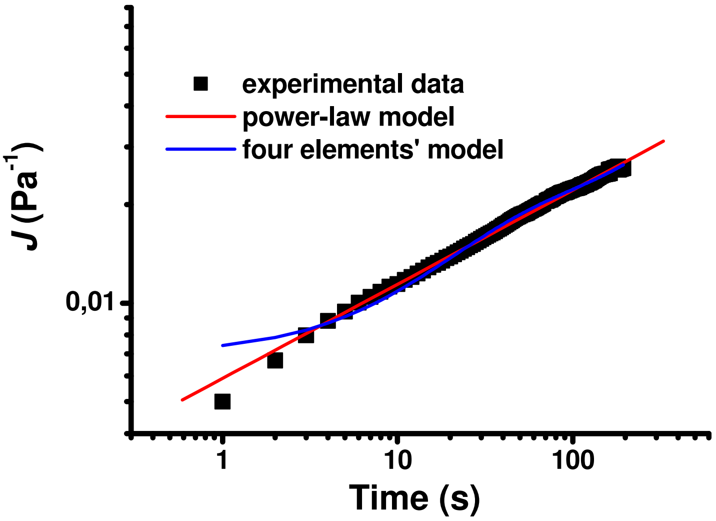

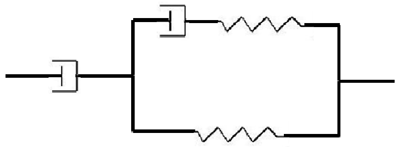

Fig. S4: Comparison of the fit to the relaxation data performed using the power-law relaxation model (red line) and the four elements' mechanical model (blue line) based on the combination of two dashpots and two springs as shown on the right. The power-law model describes better the cell tongue relaxation.

Different behaviors observed in the case of control cells

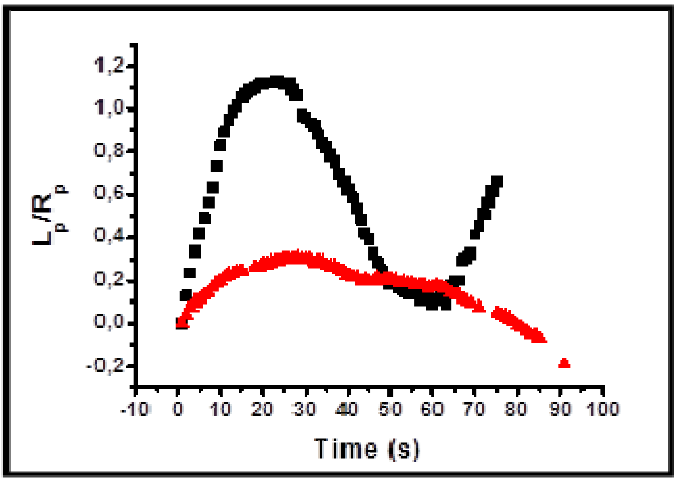

Fig. S5: Alternative behaviors, besides the power-law one, for control cells undergoing a negative pressure step of 30 Pa. The black squares show protrusion retraction after the initial fast increase followed by a new increase. Red triangles show a small protrusion increase followed by a continuous retraction.

Handling of bleb formation in the image analysis

For the behavior analysis we considered always the position of the most internal part of the cell protrusion both in the absence and in the presence of evident bleb formation. Small blebs, which are produced more easily in the case of cells treated with OTC, do not change in a substantial way the overall behavior of the cell protrusion movement. Cells forming small blebs are typically included in the case of Figure 2d. Large blebs formation confer a specific behavior to the cell protrusion (Figure 2c) characterized by a very rapid increase of the cell protrusion followed by a slow regression. After the automatic detection of the cell protrusion movement we checked anyway all the images to identify the presence of blebs such as in the case of Figure S6.

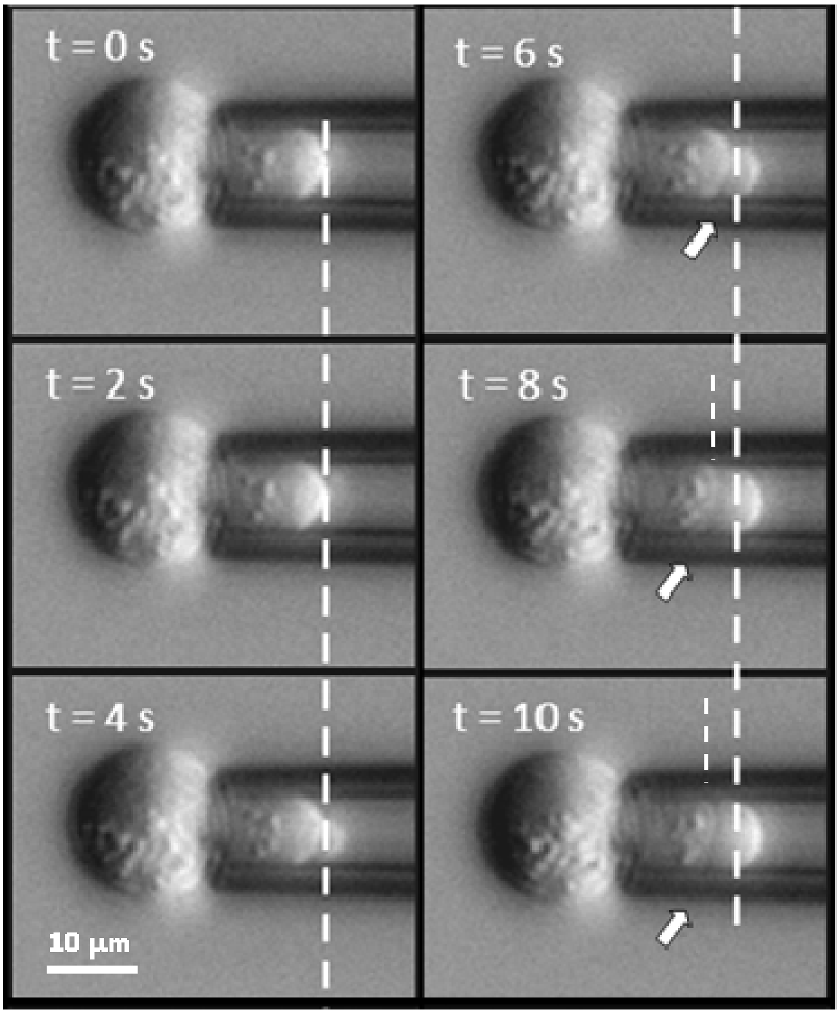

Figure S6: Example of bleb formation. We extracted a sequence of images during cell aspiration, while keeping the applied pressure constant. We choose time t = 0 s for the first image as the reference time. At time t = 4 s a membrane bleb starts to form and it grows in the following images. During the bleb growth it is possible to see that the cortical layer defining the limit of the viscous cytoplasm starts to retract (comparison between the large dashed white line and the thinner dashed white lines – see arrows). This is due to the lack of force transmission by the plasma membrane.

Cell behavior at short time

We analyzed the behavior at short time of cells showing an oscillatory behavior on a longer time scale. Here we report some of the analysis for specific incubation times. The value of the obtained α exponent is greater than the value obtained for cells showing an overall power-law behavior. Also the stiffness parameter is increased.

a

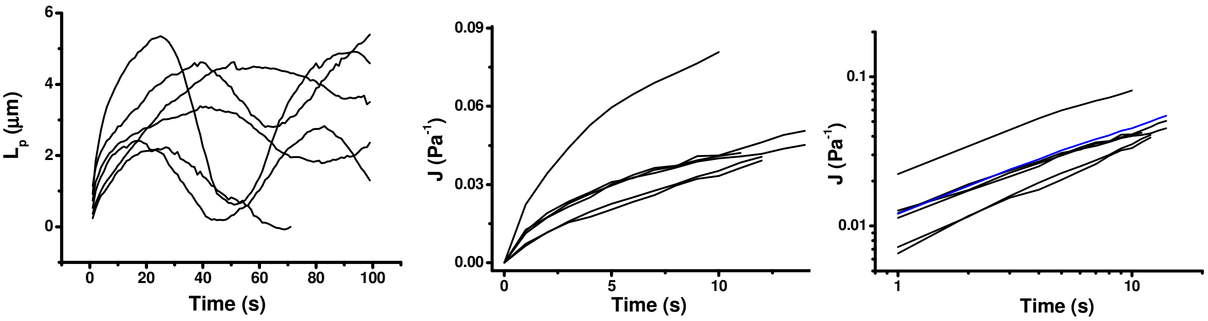

6h

b

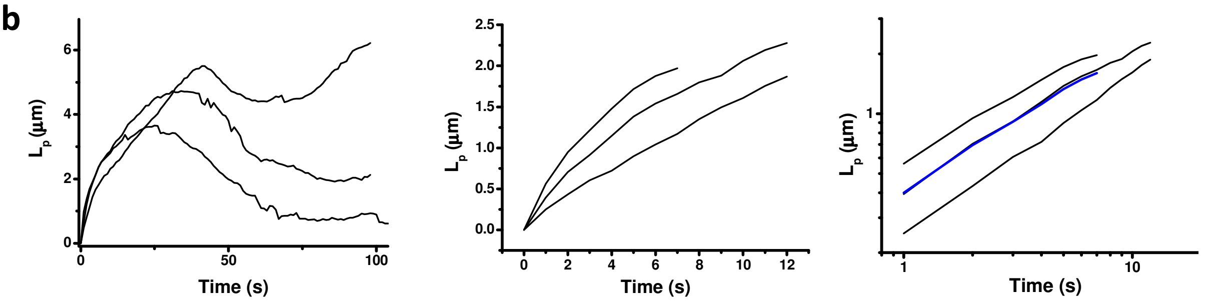

12h

c

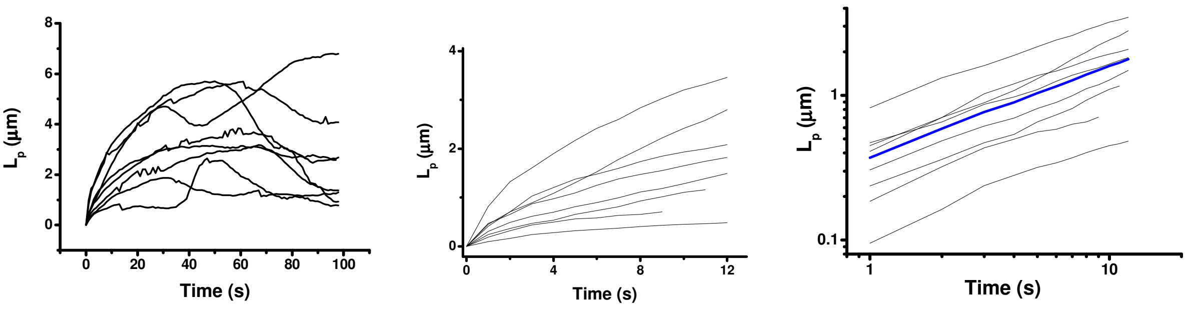

16h

Fig. S7 Analysis of the short time (from 0 s to about 15-20 s) behavior of cells showing an oscillatory behavior on the long time scale in the cases of 6h (a), 12h (b) and 16h (c) of incubation with OTC. In each case, the first plot shows the long time oscillatory behavior, the second and the third plots report the behavior of the cell tongue in the linear plot and in the Log-Log representation, respectively. In the third plot of each line, the blue line represents the averaged behavior. At short time the cells show a power-law behavior and the alpha exponent is characterized by a high value (in the range between 0.6 and 0.75) in all cases.

	Type of behavior for the cell protrusion			
Incubation time with OTC (h)	Immediate retraction	Oscillation (Cases a) and c) in Fig.2)	Power-law	Saltatory (equilibrium followed by a new increase)
(20) 0		(3) 15%	(12) 60%	(5) 25%
(20) 6	(1) 5%	(6) 30%	(8) 40%	(5) 25%
(19) 9	(3) 16%	(10) 52%	(4) 21%	(2) 11%
(20) 12		(3) 15%	(6) 30%	(11) 55%
(21) 16	(1) 4%	(8) 38%	(2) 9%	(10) 49%
(19) 18	(1) 5%	(4) 21%	(4) 21%	(10) 51%
(20)24	(2) 12%	(4) 21%	(6) 30%	(8) 37%
(21)48	(4) 18%	(3) 15%	(6) 30%	(8) 37%

Tab. S1: Number (n) and percentage of cells following the different behaviors for different OTC incubation times. The first column reports both the number of cells analyzed and the incubation time. Immediate retraction means that the cell tongue retracts almost immediately after the pressure jump is applied; oscillation means an alternating progression and retraction of the cell protrusion. In this case there is no distinction between the formation of blebs or not. This is also due to the fact that in some cases membrane detachment could occur but it could remain below the microscope resolution power; power law behavior means that the cell protrusion is continuously increasing; saltatory type means an increase of the cell projection followed by an equilibrium position which is then followed by a new increase but with no apparent bleb formation.

OTC incubation time	α exponent	Stiffness parameter (Pa)
(32) 0h (control)	0.44 ± 0.02	90 ± 6
(57) 24h	0.49 ± 0.02	101 ± 11
(60) 48h	0.57 ± 0.03	160 ± 23

Tab. S2: values of the exponent α and stiffness parameter (mean ± standard error of mean) for K562 cells exposed to OTC for different incubation times. (#): number of cells considered.

Distributions of the α value and stiffness parameter

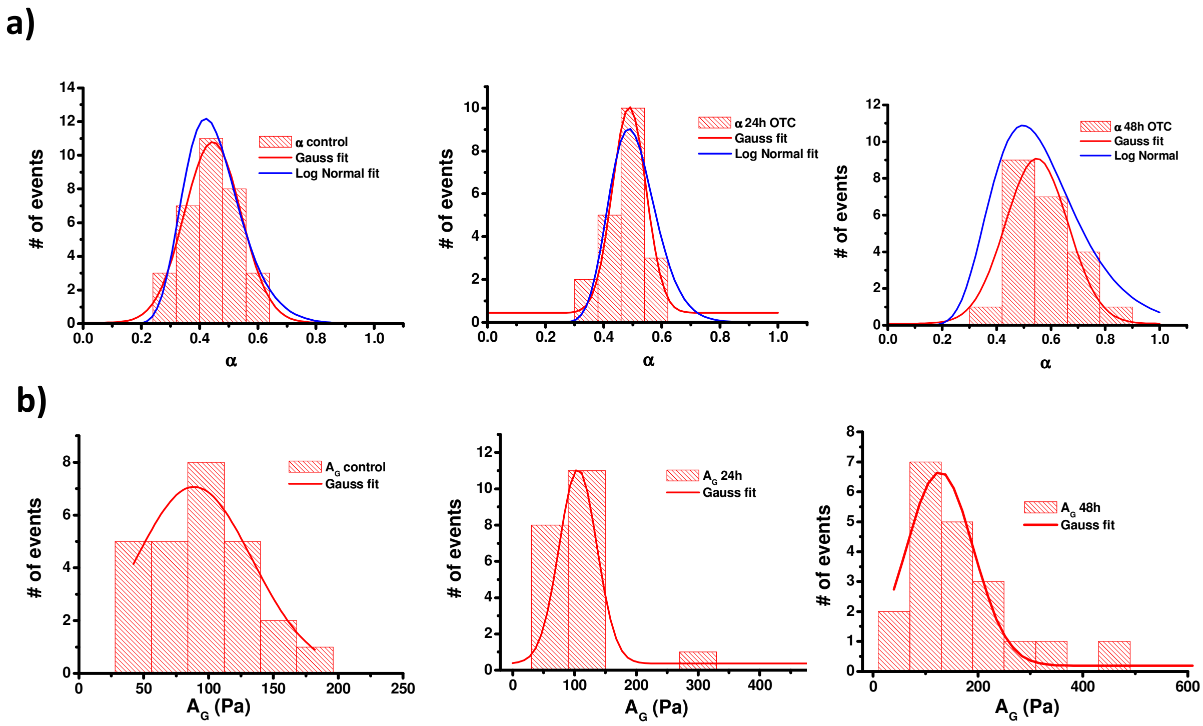

Fig. S8: a) Distribution of the α parameter for control cells, cells incubated for 24h and 48h with OTC. The distributions have been fitted by a Gaussian behavior and a Log-Normal behavior. From the fits it is not clear which is the type of distribution that fits better the data. Anyway, we used the statistical analysis for data not following a Gaussian distribution because in the literature, in many cases, it has been found that cell mechanical parameters follow a Log-Normal distribution. a) Distribution of the stiffness parameter A_G for control cells, cells incubated for 24h and 48h with OTC. The distributions have been fitted by a Gaussian behavior. The fit with the Log-Normal distribution is not good due to the limited number of points

Analysis of the correlation of the stiffness parameter or cell diameter with the fluidity parameter α

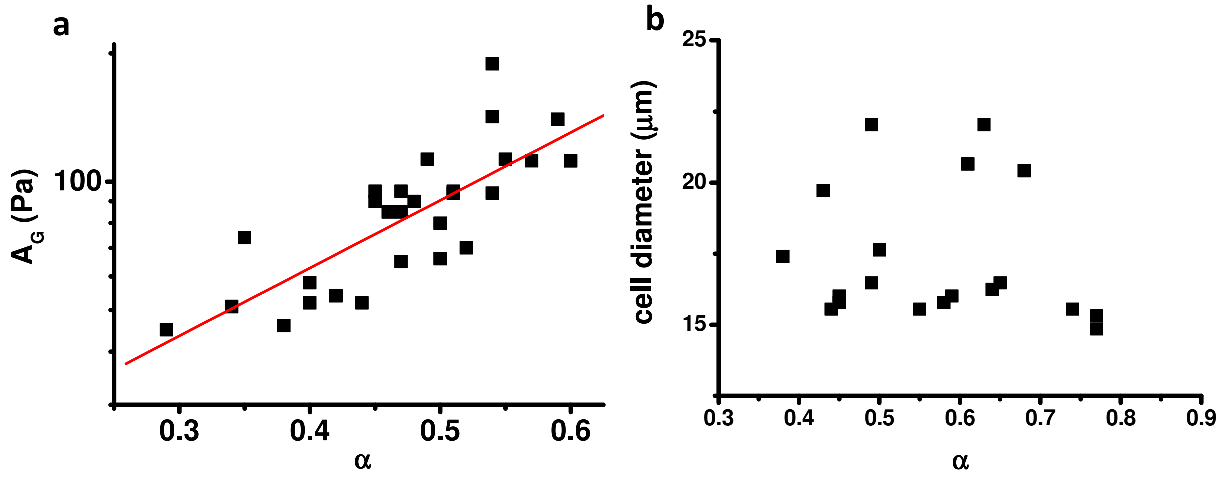

Fig. S9: a) Correlation between the A_G parameter and the α exponent for control cells ; b) correlation between the cell diameter and the corresponding alpha exponent for control cells incubated for 48 h with OTC (no correlation is found)

Examples of the obtained curves for different incubation times with OTC

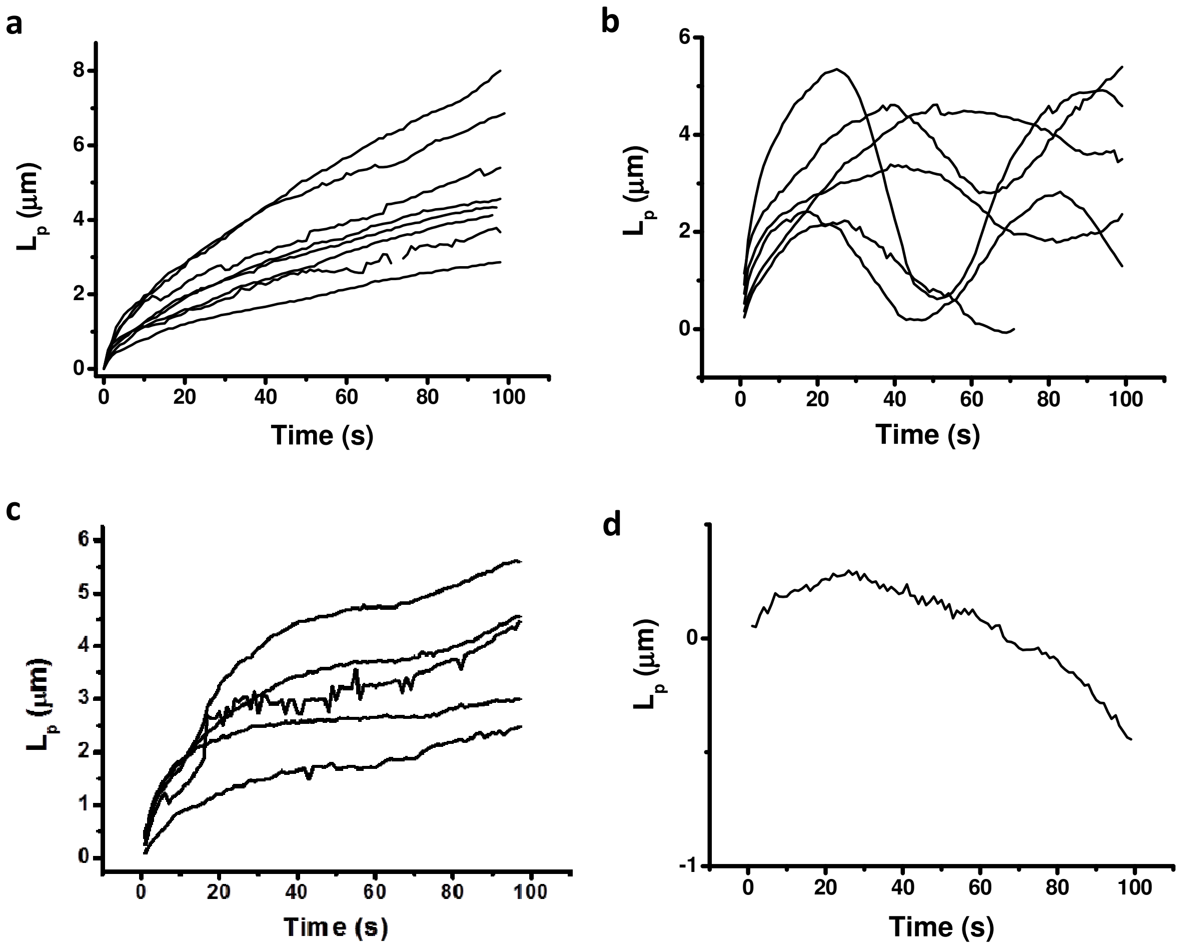

Figure S10: Curves obtained after 6h of incubation of K562 cells with OTC. Cells showing a power law behavior a), an oscillatory behavior b), a saltatory behavior c), and a cell that retracts almost immediately after the application of the pressure step d).

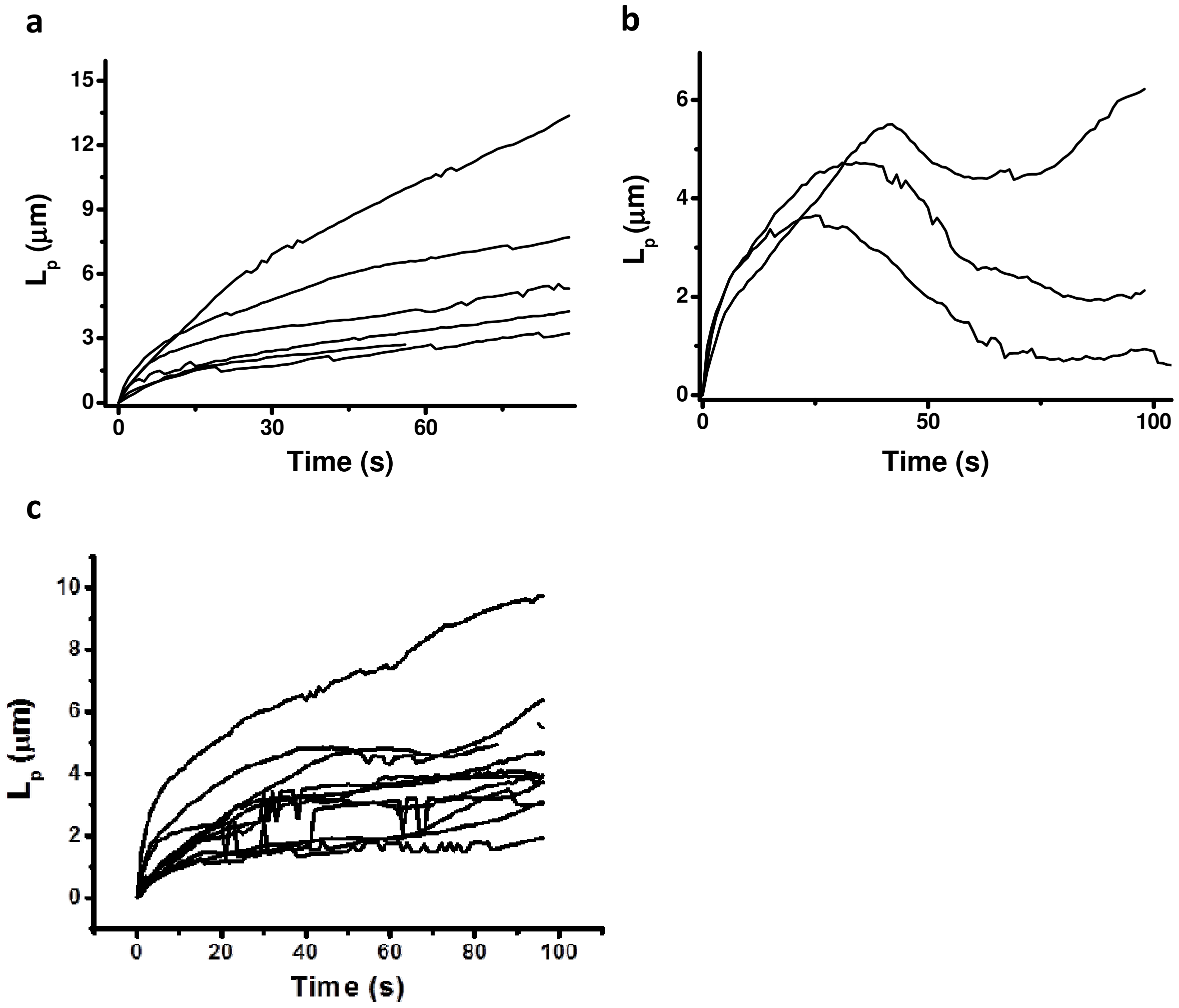

Fig. S11: Curves obtained after 12h of incubation of K562 cells with OTC. Cells showing a power law behavior a), an oscillatory behavior b) and cells with a saltatory behavior c).

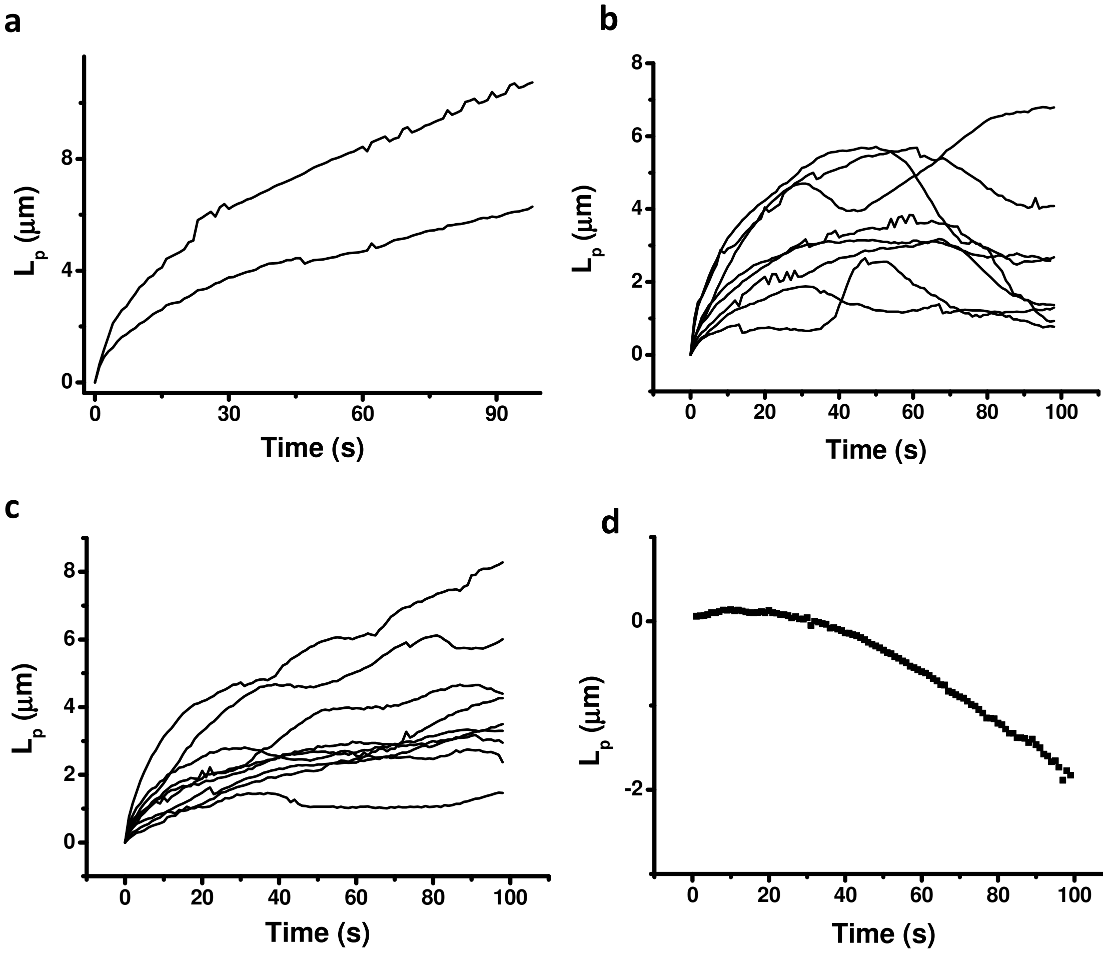

Fig. S12: Curves obtained after 16h of incubation of K562 cells with OTC. Cells showing a power-law behavior a), an oscillatory behavior b), cells with a saltatory behavior c), and a cell that retracts almost immediately after the application of the pressure step.

Permanence of oscillatory behavior after the first aspiration

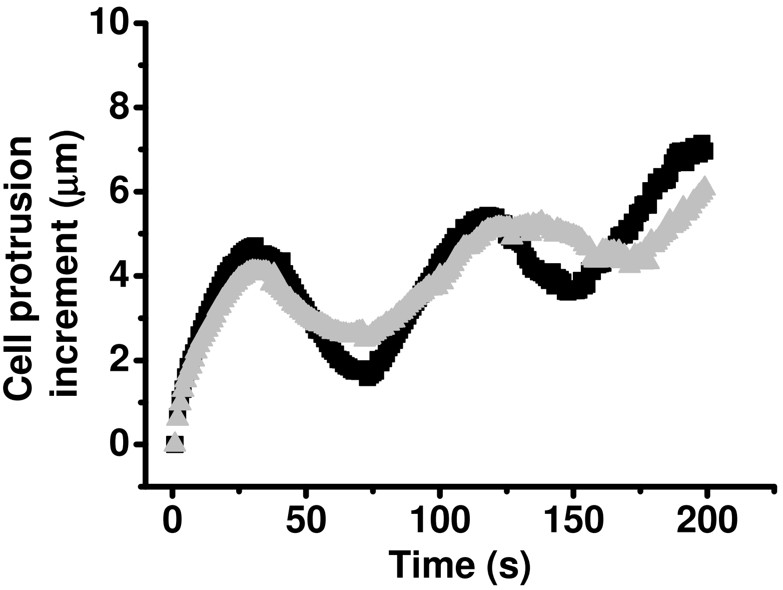

Figure S13: two consecutive applications of the same negative pressure step (black points: first run; gray dots: second run)on a cell incubated for 12h with OTC. The figure shows that the oscillating behavior is not limited to the first aspiration run. After the pressure step has been removed we waited 5 minutes to let the cell completely recover from the first aspiration.

Effect of blebbistatin on cells presenting an oscillatory behavior

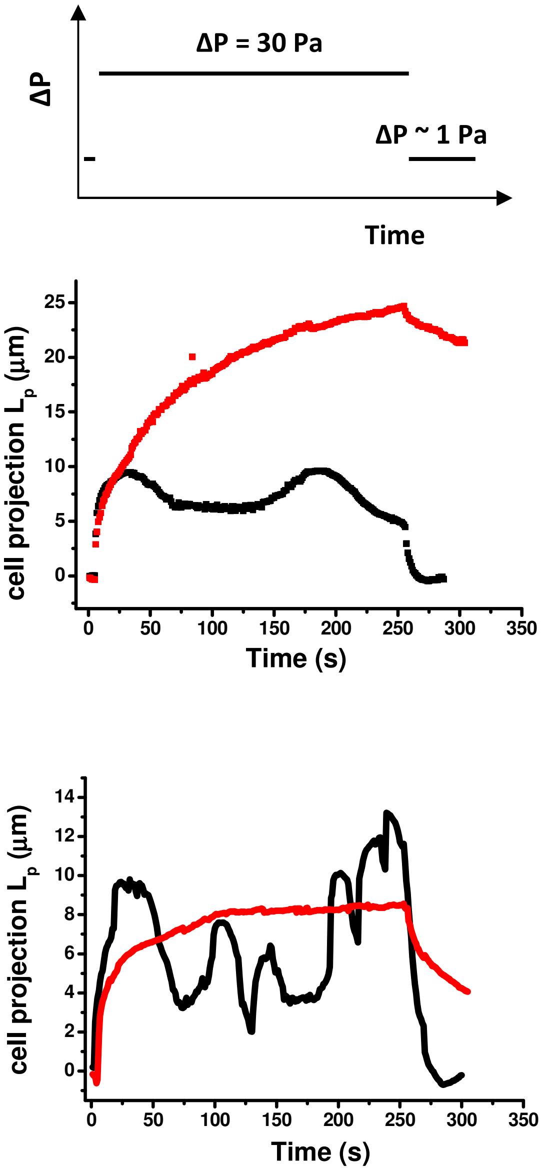

Figure S14: Two examples (another example is reported in the manuscript) of cells showing an oscillatory behavior (black traces) which is converted to a power-law behavior (red traces) after exposing the cells to 50 µM blebbistatin. Both cells were incubated with OTC for 15 h. The aspiration pressure was 30 Pa from t = 5 s to t = 250 s. After 250 s the aspiration pressure was released to a value around 1 Pa (see scheme at the top of the Figure).

Oscillatory behavior for cells incubated for 16 h with OTC + blebbistatin

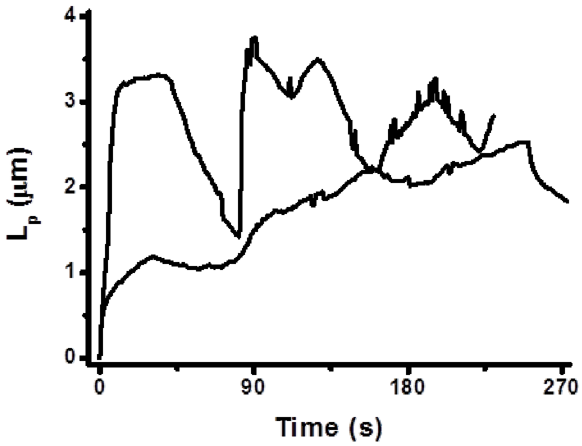

Fig. S15: example of the oscillatory behavior for cells which were incubated with OTC+blebbistatin and then aspirated in the absence of blebbistatin.

Movies representing the different observed cell tongue behaviors

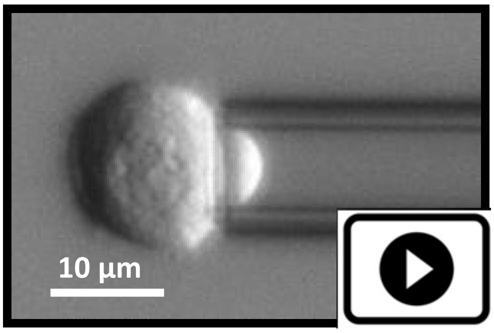

Movie S1: Cell protrusion growth following the power-law relation. The movie shows a sequence of optical microscopy images acquired in the DIC mode for a cell not exposed to OTC and subjected to a pressure jump of 30 Pa. The time interval between two consecutive images is 1 second (in the movie the time sequence is accelerated).

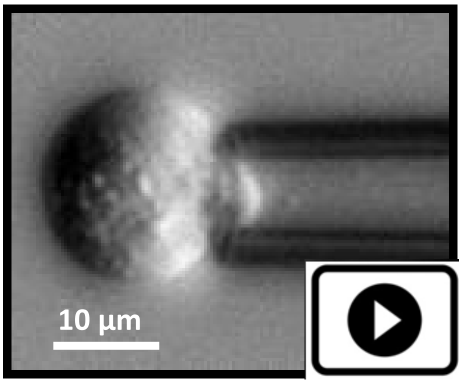

Movie S2: Sequence of the position of the protrusion of a cell which was exposed for 9h to OTC. The occurrence of very rapid forward steps associated with cell blebbing is evident. Each forward step is then followed by a slow protrusion retraction probably due to cortical actin re-assembly. The time interval between two consecutive images is 1 second (in the movie the time sequence is accelerated).

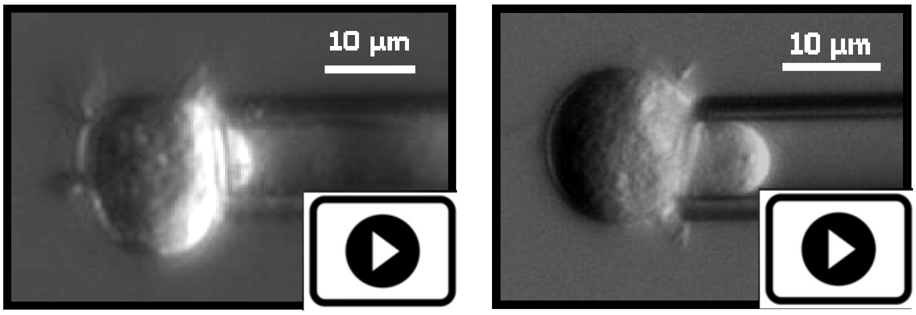

Movie S3: a and b) Movies of a cell exposed for 16h to OTC and then subjected to a constant negative pressure of 30 Pa. The cell protrusion exhibits oscillations while kept at constant pressure. When cells like the ones represented in these movies were exposed to 50 µM blebbistatin the oscillation behavior was converted into a continuous growth of the protrusion. The time interval between two consecutive images is 1 second (in the movie the time sequence is accelerated).

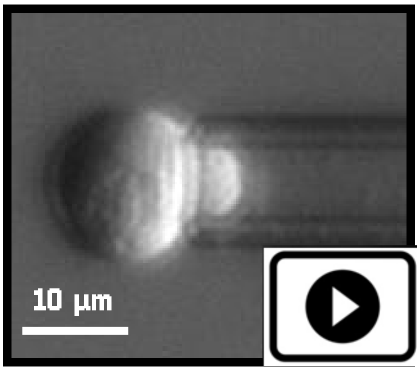

Movie S4: Example of a cell exposed for 12 h to OTC and showing a saltatory behavior consisting in an increase of the protrusion followed by a stationary phase and then by a new increase. The time interval between two consecutive images is 1 second (in the movie the time sequence is accelerated).

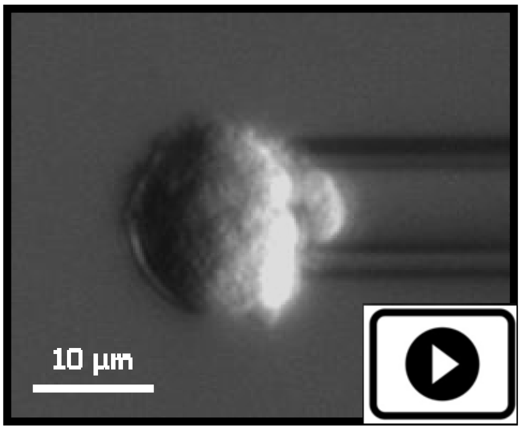

Movie S5: Movie of a K562 cell that was exposed for 48 h to OTC. After the application of the negative pressure jump of 30 Pa the cell protrusion starts to retract and at the end blebs are formed on the external portion of the cell. The time interval between two consecutive images is 1 second (in the movie the time sequence is accelerated).
